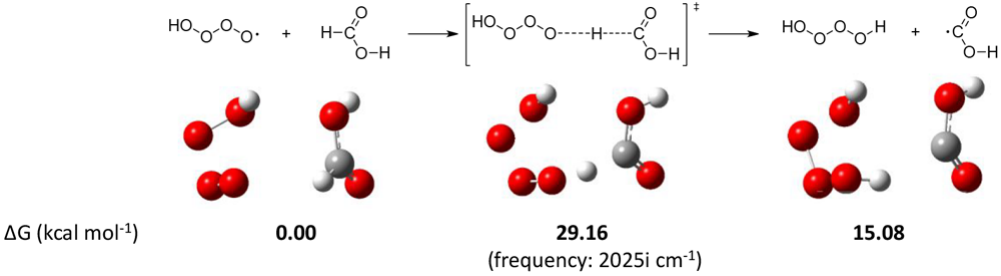# Mechanistic Insights into the Oxidative Degradation of Formic and Oxalic Acids with Ozone and OH Radical. A Computational Rationale

**DOI:** 10.1021/acs.jpca.2c08091

**Published:** 2023-02-07

**Authors:** Fernando J. Beltrán, Ana María Chávez, Pedro Cintas, R. Fernando Martínez

**Affiliations:** † Departamento de Ingeniería Química y Química Física, Facultad de Ciencias, and Instituto Universitario de Investigación del Agua, Cambio Climático y Sostenibilidad, (IACYS), Universidad de Extremadura, Avenida de Elvas s/n, 06006 Badajoz, Spain; ‡ Departamento de Química Orgánica e Inorgánica, Facultad de Ciencias, and Instituto Universitario de Investigación del Agua, Cambio Climático y Sostenibilidad, (IACYS), Universidad de Extremadura, Avenida de Elvas s/n, 06006 Badajoz, Spain

## Abstract

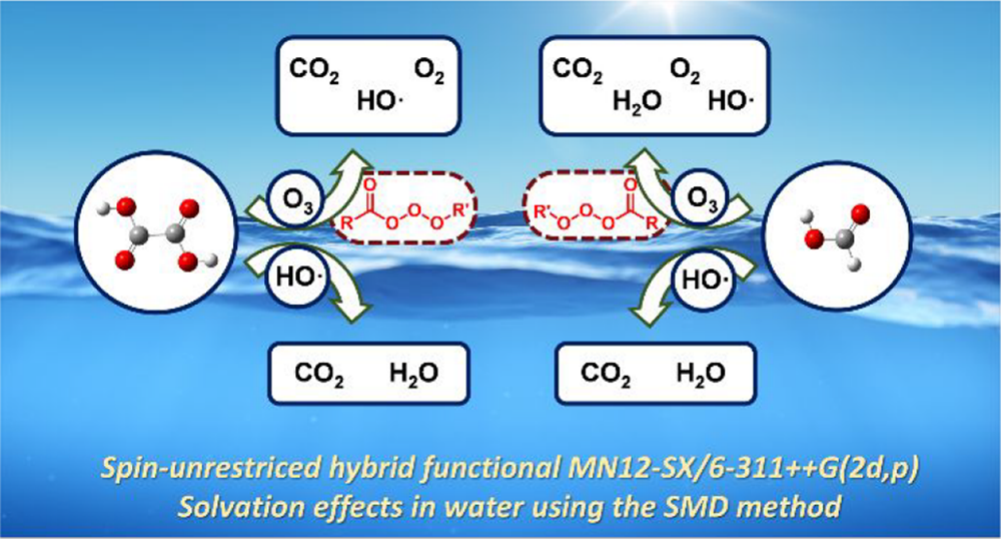

Gas-phase and aqueous oxidations of formic and oxalic acids with ozone and OH radicals have been thoroughly examined by DFT methods. Such acids are not only important feedstocks for the iterative construction of other organic compounds but also final products generated by mineralization and advanced oxidation of higher organics. Our computational simulation unravels both common and distinctive reaction channels, albeit consistent with known H atom abstraction pathways and formation of hydropolyoxide derivatives. Notably, reactions with neutral ozone and OH radical proceed through low-energy concerted mechanisms involving asynchronous transition structures. For formic acid, carbonylic H-abstraction appears to be more favorable than the dissociative abstraction of the acid proton. Formation of long oxygen chains does not cause a significant energy penalty and highly oxygenated products are stable enough, even if subsequent decomposition releases environmentally benign side substances like O_2_ and H_2_O.

## Introduction

Ozonation has become a valuable and efficient tool in disinfection treatments of drinking water and wastewater, resulting from the rapid degradation of numerous organic pollutants^1,2^ and inactivation of pathogenic microorganisms^3,4^ present in aqueous environments. Like other advanced oxidation processes (AOPs), ozone-based protocols can be conducted alone and in combination with a broad range of activation methods, ranging from photoirradiation and the use of both homogeneous and heterogeneous catalysts.^5−10^ In general, ozone is more efficient than molecular oxygen in the catalytic oxidation of volatile organic compounds (VOCs), and higher conversions can be achieved at lower temperatures and mild conditions.^5^

The aqueous chemistry of ozone-mediated reactions may involve intermediates and radical species observed in gas-phase reactions, widely studied in the context of ozone depletion or atmospheric aerosol formation.^11−14^ However, additional and distinctive reaction channels arise from phenomena such as solvation, ionization, and intermolecular associations, which complicate the search of satisfactory interpretations. Thus, although ozonation is extremely useful from a practical viewpoint, there is still a lack of mechanistic understanding at the molecular level, and the putative pathways, especially with complex organics, remain speculative. In recent years, application to AOPs of first-principle calculations, often aided by density functional theory (DFT) simulations, sheds light upon reaction pathways and intermediates, which have eventually been identified by experiments. Among them, Criegee intermediates (carbonyl oxides), produced by the ozonolysis of unsaturated hydrocarbons in the atmosphere, can also trigger subsequent reactions with both inorganics and organics and leading to aerosol formation.^15−18^

Herein, we provide an in-depth computational study on the interaction of ozone with formic and oxalic acids, which are archetypal ozonation reactions assessed by numerous experimental analyses, and serving as reactivity models of ozone toward organic acids. Moreover, such low-mass C1 and C2 carboxylic acids are often end-products detected during AOPs of other higher organic pollutants.

The reaction of ozone with formic acid in aqueous solution should be fairly credited to Nobelist Henry Taube, who in the early 1940s showed the existence of a chain reaction involving the formation of HO–HO_2_ species accounting for the decomposition of ozone and correlating well with results obtained in the ozone–peroxide reactions.^19^ As mentioned, formic acid is a common product found in the ozonolysis of aliphatic and substituted aromatic organics, often enhancing the oxidation when used as solvent. Combination of ozone–formic acid and H_2_O_2_–formic acid have long been used as oxidative degradation methods, and the intermediacy of performic acid was invoked more than 60 years ago.^20^ To a minor extent, oxalic acid is also present as a decomposition product.^21^ On the other hand, there has been a plethora of kinetic data on the OH-induced decomposition of organic acids, although rates reported should be interpreted with caution because experimental conditions vary from paper to paper.^22^ For ionizing compounds, the second-order rate constants increase with pH as does deprotonation of the dissolved acid.^23^ This also reflects the conversion of ozone into OH radicals by reaction with hydroxyl anions.^24^ However, the dependence of rate constants on pH lacks a clear-cut relationship when OH radicals are directly evaluated instead of ozone.^22^ In general, when the oxidation of organic compounds affords oxalic or acetic acid, further mineralization to CO_2_ is slower than oxidations leading to formic acid or formate.^23,25^ Remarkably, the presence of formate ions causes significant acceleration of oxidation, presumably due to formation of carbonate radical anion (CO_3_^•–^) as evidenced by the use of radical probes.^26^ Such an intermediate appears to occur in the gas-phase oxidation of formic acid with ozone, though mediated by tropospheric electrons.^27^ Despite variations, rate constants obtained by radiolytic^28^ and photochemical methods exhibit good agreement^21,29^ and are substantially enhanced when catalysts are present, thereby showing the synergetic effect owing to production of OH radicals.^30−32^

Although structural information cannot be inferred from kinetic data, it is evident the distinctive behavior of the actual oxidative agents, which depend in turn on the initial conditions. With these premises and in line with the scope of this theoretical study, reactions of formic and oxalic acids with either ozone or hydroxyl radicals have been computed leading to saddle points and products, whose structure, energy barriers, and stability are also consistent with the apparent rapidity observed by experimental methods.

## Computational Methods

All calculations were carried out using the Gaussian16 package.^33^ Geometries and energies were optimized using the spin-unrestricted hybrid functional MN12-SX,^34^ which is a range-separated-hybrid meta-NGA (nonseparable gradient approximation),^35^ in combination with the Pople 6-311++G(2d,p) basis set.^36^ Geometries were optimized with inclusion of solvation effects in water using the SMD method.^37,38^ All saddle points linking specific reactant and products through the reaction path were verified using intrinsic reaction coordinate (IRC) analysis. Frequency calculations were carried out at 298.15 K at the above-mentioned level of theory. Saddle points and energy minima were characterized by one or none imaginary frequencies, respectively.

## Results and Discussion

### Reaction of Formic Acid and Ozone

**Scheme 1:**
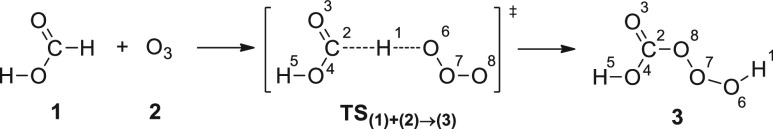
Abstraction of the Carbonylic H-Atom of Formic Acid by Ozone

Formic acid exemplifies well the case of double-H atom abstraction (or transfer) reactions, which are of paramount importance in atmospheric and aquatic chemistry. While numerous organic compounds are sensitive to single hydrogen transfer upon reaction with the OH radical, carboxylic acids are also prone to undergo both hydration and dehydration reactions.^39^ Organic acids containing α-hydrogens are susceptible to hydrogen transfer at the acidic carboxyl moiety and at alkyl carbons. In formic acid, the latter occurs at a less hindered sp^2^-hybridized carbon. This process is feasible enough with neutral O_3_ and our theoretical simulation is displayed in Scheme 1 (for the sake of clarity, atom numbering is shown along the different structures). Frontal attack of one terminal oxygen of ozone (O^6^ in Scheme 1) to aldehydic H atom of formic acid (H^1^) leads to the saddle point **TS**_**(1)+(2)→(3)**_, which features similar geometric characteristics in both the gas phase and water, namely, the bond lengths H^1^–O^6^ (1.17 Å in the gas phase and 1.20 Å in water) and H^1^–C^2^ (1.36 Å in the gas phase and 1.34 Å in water), together with the angle C^2^–H^1^–O^6^ (168° in the gas phase and 174° in water) and dihedral angle H^1^–O^6^-O^7^–O^8^ (−60° in the gas phase and −72° in water). As expected, the saddle points were unambiguously characterized by their imaginary frequencies, at 1837i cm^–1^ (gas phase) and 1873i cm^–1^ (water).

As usual, IRC (intrinsic reaction coordinate) analysis is customary to trace the path from the TS to the products and reactants. However, the curvature and length of the path also provides some insights into the reaction itself. Here, the IRC of **TS**_**(1)+(2)→(3)**_ (Figure S1) evidence that the H^1^–C^2^ bond breaking is accompanied by a progressive shortening between the O^8^ and C^2^ atoms, thereby affording in concerted fashion the trioxidane derivative, **3**, which may be regarded as the insertion product of ozone into the C–H bond of formic acid.

Formation of the highly oxygenated product **3** is consistent with previous literature reporting the chemistry of hydrogen trioxide, HOOOH, called *trioxidane* according to IUPAC nomenclature, which is the simplest polyoxide of formula RO_*n*_R (*n* ≥ 3) with R standing for hydrogen as well as alkyl, acyl, or other atoms.^40,41^ These elusive species are generated by ozonation of carbon–hydrogen bonds, i.e., from a formal viewpoint by the reduction of ozone by activated C–H bonds. Surprisingly, organic hydrotrioxides (ROOOH), which are indeed strong oxidants, have long been omitted in atmospheric chemistry, whereas a recent study evidences their formation by combination of peroxy radicals (RO_2_) and hydroxyl radicals (OH) (*vide infra*). In fact, this appears to be a dominant pathway in the atmosphere starting from volatile terpenes and hydrocarbons, from which peroxy species can easily be generated.^42^ Other mechanistic surrogates have been proposed in the last four decades, ranging from a 1,3-dipolar insertion of ozone to radical pairs and hydride ion abstraction.^41^ A better picture suggests that H atom abstraction and hydride abstraction would actually be extreme situations between a reaction complex with bifurcations depending on the experimental conditions.^43^

**Scheme 2:**
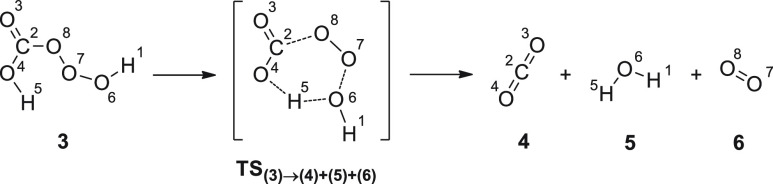
Evolution of Trioxide 3 to Compounds 4–6

Trioxide **3** evolves through the shortening and subsequent transfer of H^5^ to O^6^ followed by C^2^–O^8^ bond breaking, which leads to the saddle point **TS**_**(3)→(4)+(5)+(6)**_ [C^2^–O^8^ 1.68 Å, O^6^–O^7^ 1.68 Å, H^5^–O^6^ 1.08 Å, H^5^–O^4^ 1.36 Å in the gas phase and C^2^–O^8^ 1.63 Å, O^6^–O^7^ 1.69 Å, H^5^–O^6^ 0.98 Å, H^5^–O^4^ 2.19 Å in water] that directly drives to naturally occurring end products, namely CO_2_ (**4**), H_2_O (**5**), and O_2_ (**6**) (Scheme 2). IRC of the saddle point **TS**_**(3)→(4)+(5)+(6)**_ (Figure S2) proves the aforementioned reaction path, and as expected, such a transition structure exhibited only one imaginary frequency (741i cm^–1^ in the gas phase and 382i cm^–1^ in water).

The reactions of unsaturated oxygen-containing compounds of atmospheric relevance have been evaluated by theoretical methods,^44^ although the role of trioxides is scarcely addressed. The putative intermediates (either acyclic or cyclic in particular) have also been the subject of controversy, albeit results with carbonyls are illuminating in context. Thus, benzaldehyde undergoes ozone insertion into the aldehydic C–H bond. DFT calculations, at the B3LYP/6-31G(d,p) level, evidence the highly exothermic formation of benzoyl hydrotrioxide, i.e., C_6_H_5_C(O)OOOH, which is conformationally unstable ending up with the elimination of benzoic acid and singlet oxygen.^45^ The stability of long oxygen chains, higher than H_2_O_2_, has been rationalized in terms of bond dissociation energies, and rapid decomposition is expected on moving from H_2_O_3_ (hydrogen trioxide) to the most reactive H_2_O_4_ and H_2_O_6_.^46^ Ozonation reactions with saturated oxygenates are less known, but they would similarly proceed through H-atom abstraction leading to formation of hydrotrioxides, which then evolve to R–OH and O_2_, according to a recent computational study performed on acyclic and cyclic oxolanes, alkyl hydroperoxides, and ethers.^47^

**Scheme 3:**
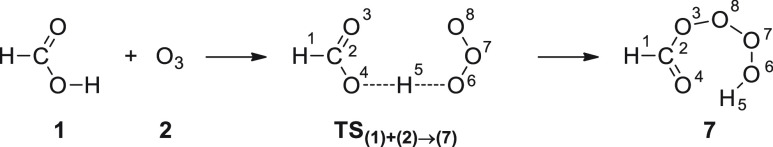
Formation of Tetraoxidane 7 by Reaction of Formic Acid and Ozone Involving Abstraction of the Acidic Proton H^5^

On the other hand, ozone can also react with formic acid by abstracting the acidic (carboxylic) proton (H^5^). Computation unravels a frontal attack as well, as shown in Scheme 3, giving rise to tetraoxidane **7**.

The approach of the O^6^ atom to H^5^ proceeds in a concerted way affording the saddle point **TS**_**(1)+(2)→(7)**_, characterized by the bond distances O^4^–H^5^ (1.23 Å in both media) and H^5^–O^6^ (1.17 Å in both media too), the angle O^4^–H^5^–O^6^ (172° in the gas phase and 173° in water), and the dihedral angle H^5^–O^6^–O^7^–O^8^ (−39° in the gas phase and −42° in water), with imaginary frequencies of 1179i cm^–1^ (gas phase) and 1232i cm^–1^ (water).

In a similar way to the preceding C–H cleavage, the IRC analysis of **TS**_**(1)+(2)→(7)**_ (Figure S3) shows that O^4^–H^5^ bond breaking occurs with the concomitant shortening of the O^8^–O^3^ distance, leading to compound **7** in a concerted manner. These results indicate that abstraction of the carbonylic (aldehydic) hydrogen by ozone is more favorable in aqueous solution than the alternative and chemoselective abstraction at the acidic moiety (ΔΔ*G*^‡^ = 2.07 kcal mol^–1^), while the gas-phase reaction follows the opposite trend, by favoring the abstraction of the carboxylic proton (ΔΔ*G*^‡^ = 1.63 kcal mol^–1^).

**Scheme 4:**
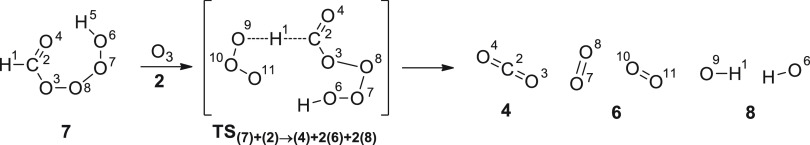
Evolution of Tetraoxidane 7 to End Species 4, 5, and 8

The evolution of tetraoxidane **7** is initiated by the attack of a new molecule of ozone (**2**) to H^1^ of **7**, which takes place through the saddle point **TS**_**(7)+(2)→(4)+2(6)+2(8)**_ [bond length H^1^–C^2^ (1.31 Å in the gas phase and 1.33 Å in water); bond length H^1^–O^9^ (1.24 Å in the gas phase and 1.21 Å in water); angle C^2^–H^1^–O^9^ (160° in the gas phase and 178° in water); imaginary frequency (1736i cm^–1^ in the gas phase and 2015i cm^–1^ in water)] in which the abstraction of H^1^ by O^9^ is accompanied by O^3^–O^8^, O^6^–O^7^, and O^9^–O^10^ bond breaking, giving rise to CO_2_ (**4**), two molecules of O_2_ (**6**), and two hydroxyl radicals (**8**) (Scheme 4). Again, IRC analysis of **TS**_**(7)+(2)→(4)+2(6)+2(8)**_ agrees with the reaction path depicted above (Figure S4).

**Figure 1:**
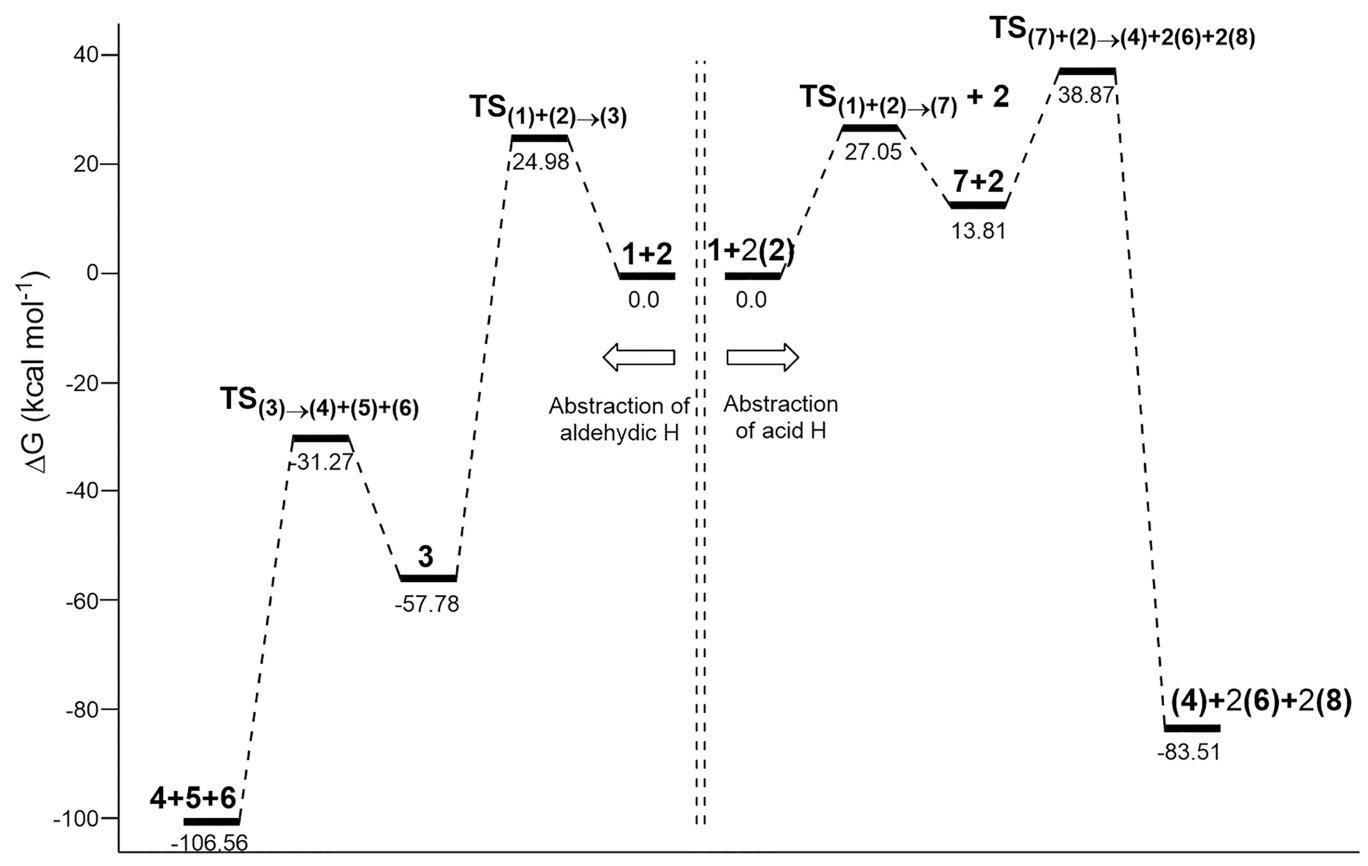
Relative free energies (Δ*G*, kcal mol^–1^) of all stationary points involved in the reaction of formic acid with ozone, at the UMN12SX/6-311++G(2d,p) level of theory with bulk solvation in water (SMD method).

Figure 1 and Figure S15 collect thermochemical data for the reaction of formic acid with ozone. This indicates that carbonylic H-abstraction is a little bit more favorable than the abstraction of the acid proton in water, but it is much less favorable than the abstraction of the acid proton in the gas phase.

### Reaction of Oxalic Acid and Ozone

**Scheme 5:**
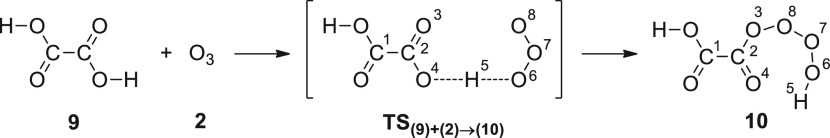
Reaction of Oxalic Acid with Ozone Forming Tetraoxidane 10

Unlike formic acid, oxalic acid (**9**) can only undergo H atom abstraction at the carboxylic fragment. The direct reaction with ozone is shown in Scheme 5.

The approach of one terminal oxygen (O^6^) to the H^5^ atom of **9** evolves with the formation of **TS**_**(9)+(2)→(10)**_ (Δ*G*^‡^ = 21.77 kcal mol^–1^ in the gas phase and 28.16 kcal mol^–1^ in water; imaginary frequencies 1039i cm^–1^ and 722i cm^–1^, respectively) leading to the neutral trioxidane derivative **10**. The key structural parameters are seldom distorted by solvation as reflected by bond distances O^4^–H^5^ (1.26 and 1.31 Å) and H^5^–O^6^ (1.15 and 1.12 Å, respectively), with angles O^4^–H^5^–O^6^ (172° in both media) and H^5^–O^6^–O^7^–O^8^ (−38° and −34°, respectively). In agreement with previous IRC analyses, this attack exhibits complete concertedness for the insertion of ozone into the O–H bond of oxalic acid (Figure S5) and, as expected, the reactivities of the carboxylic hydrogen atom in both formic and oxalic acids are similar, irrespective of the environment considered (ΔΔ*G*^‡^ = 0.22 kcal mol^–1^ in the gas phase and 1.11 kcal mol^–1^ in water).

**Scheme 6:**
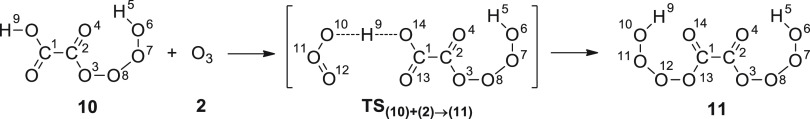
Reaction of Tetraoxidane 10 with O_3_ to Give a Hydropolyoxidane Structure

The attack of a second ozone molecule (**2**) to H^9^ of **10** leads to saddle point **TS**_**(10)+(2)→(11)**_ [bond distance H^9^–O^14^ (1.27 Å in the gas phase and 1.36 Å in water); bond distance H^9^–O^10^ (1.14 Å in the gas phase and 1.09 Å in water); angle O^14^–H^9^–O^10^ (172° in both media); imaginary frequencies (927i cm^–1^ in the gas phase and 337i cm^–1^ in water)] in which the abstraction of H^9^ by O^10^ is accompanied by the formation of the O^12^–O^13^ bond (Scheme 6, Figure S6).

**Scheme 7:**
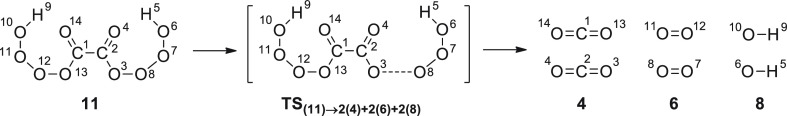
Decomposition of Bis(tetraoxidane) 11 to CO_2_, O_2_, and HO^•^

The decomposition of bis(tetraoxidane) **11** is a fast process starting with O^4^–O^8^ bond breaking and followed by the progressive scission of C^1^–C^2^, O^12^–O^13^, O^6^–O^7^, and O^10^–O^11^ bonds to form CO_2_ (**4**), two molecules of O_2_ (**6**), and two hydroxyl radicals (**8**) through the saddle point **TS**_**(11)→(4)+2(6)+2(8)**_ [bond length O^3^–O^8^ (2.21 Å in the gas phase and 2.33 Å in water); imaginary frequencies (259i cm^–1^ in the gas phase and 215i cm^–1^ in water)] (Scheme 7, Figure S7).

**Figure 2:**
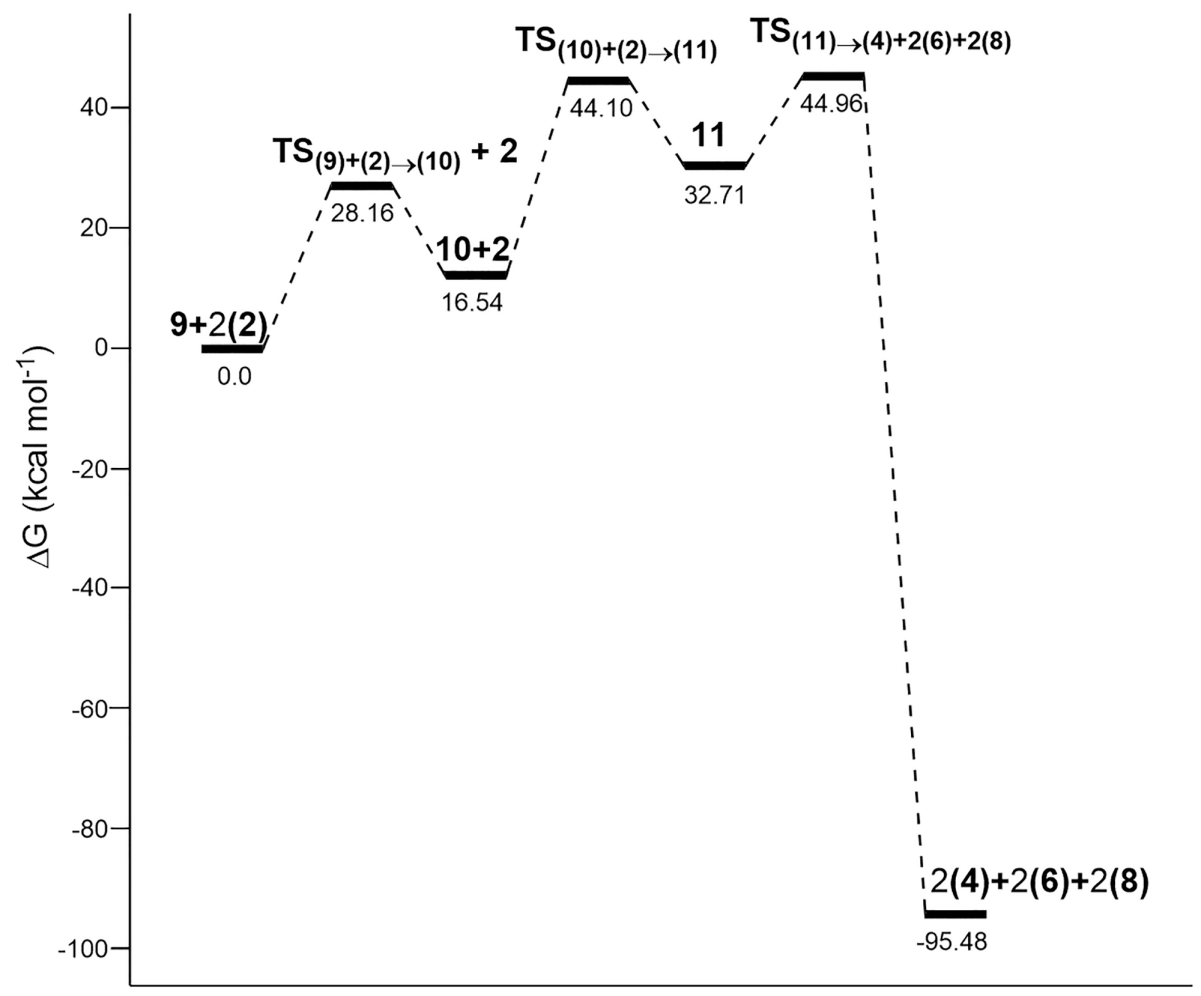
Relative free energies (Δ*G*, kcal mol^–1^) for all stationary points involved in the reaction of oxalic acid with ozone, at the UMN12SX/6-311++G(2d,p) level with bulk solvation in water (SMD method).

Figure 2 and Figure S16 gather thermochemical data for the reaction of oxalic acid (**9**) with ozone (**2**). Computational simulations show that the decomposition of the bis(tetraoxidane) **11** takes place with similar energy barriers in both the gas phase and water (∼12 kcal mol^–1^).

### Reaction of Oxalic and Formic Acids with OH Radical

**Scheme 8:**
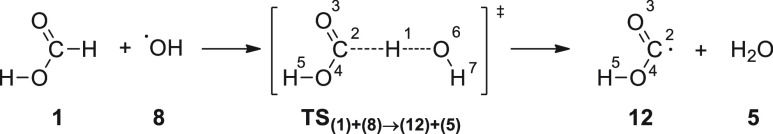
Reaction of Formic Acid with OH Radical

As mentioned in our introductory remarks, the oxidation of formic (**1**) and oxalic (**9**) acids with transient OH radicals generated by different AOP protocols, instead of the direct condensation with net ozone, has been taken into account for comparative purposes. The first step involves the reaction of HO^•^ (**8**) with the carbonylic atom (H^1^) of HCOOH (**1**) (Scheme 8, Figure S8) yielding the radical species **12**. The abstraction reaction of H^1^ by the hydroxyl radical is a fast step that proceeds through the formation of saddle point **TS**_**(1)+(8)→(12)+(5)**_ (Δ*G*^‡^ = 8.51 kcal mol^–1^ in the gas phase and 10.33 kcal mol^–1^ in water). The structural parameters affected involve the H^1^–O^6^ (1.38 and 1.42 Å, respectively) and H^1^–C^2^ distances (1.19 and 1.18 Å, respectively), together with the angle C^2^–H^1^–O^6^ (162° and 170°, respectively) and have a lower imaginary frequency than those of ozonation reactions (582i cm^–1^ in the gas phase and 556i cm^–1^ in water).

**Scheme 9:**
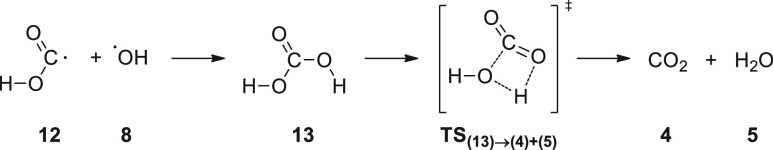
Transformation of Radical 12 into CO_2_ and Water

Once radical **12** is formed, the collision of a new OH radical (**8**) with **12** generates carbonic acid (**13**) through a barrierless process, which is further transformed into CO_2_ and water via saddle point **TS**_**(13)→(4)+(5)**_ (Scheme 9, Figure S9).

**Scheme 10:**
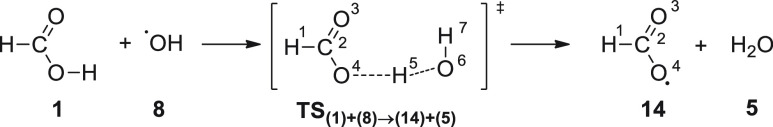
Abstraction of Acid Proton H^5^ of Formic Acid by OH Radical

**Figure 3:**
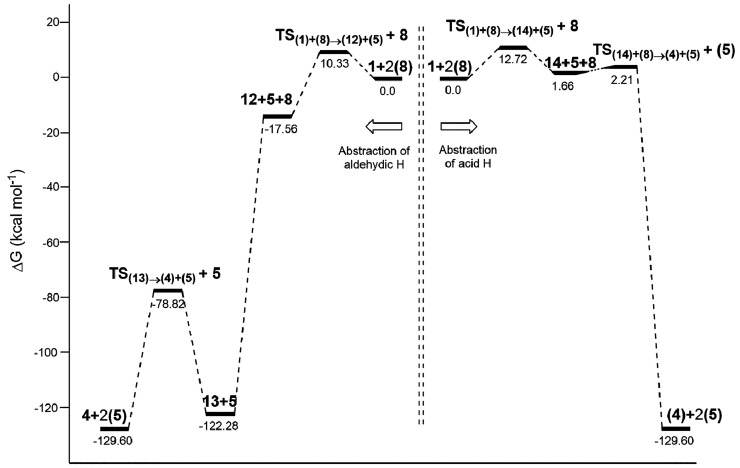
Relative free energy values (Δ*G*, kcal mol^–1^) of all stationary points involved in the reaction of formic acid with OH radical, at the UMN12SX/6-311++G(2d,p) level with inclusion of solvation effects in water (SMD method).

Alternatively, the reaction of OH radicals at the carboxylic acid terminus is shown in Scheme 10. The first step in which a reactive species (**8**) reacts with the H^5^ atom of HCOOH (**1**) produces another radical (**14**). As expected for this free-radical transformation, the saddle point **TS**_**(1)+(8)→(14)+(5)**_ is quickly reached (Δ*G*^‡^ = 7.79 kcal mol^–1^ in the gas phase and 12.72 kcal mol^–1^ in water, as displayed in Figure 3 and Figures S10 and S17). Consistent with the different geometries of the aldehydic and carboxylic moieties, the latter now shows shorter H^5^–O^6^ (1.21 and 1.22 Å, respectively) and O^4^–H^5^ (1.20 and 1.19 Å, respectively) distances with characteristic angles O^4^–H^5^–O^6^ (155° and 156°) and O^4^–H^5^–O^6^–H^7^ (84° and 86°, respectively).

**Scheme 11:**
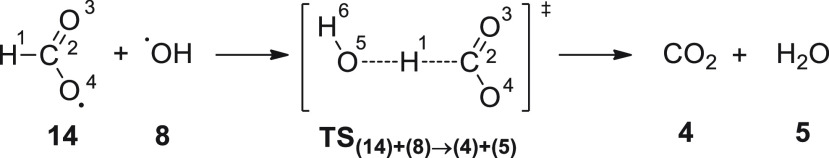
Evolution of Radical 14 to CO_2_ and H_2_O

Next, the attack of a new OH radical (**8**) to hydrogen H^1^ of **14** leads to CO_2_ (**4**) and H_2_O (**5**) via saddle point **TS**_**(14)+(8)→(4)+(5)**_, characterized by H^5^–O^1^ (1.39 Å in the gas phase and 1.34 Å in water) and H^1^–C^2^ (1.27 Å in the gas phase and 1.30 Å in water) bond distances, angle O^5^–H^1^–C^2^ (128° in the gas phase and 131° in water), dihedral angle H^6^–O^5^–H^1^–C^2^ (−75° in the gas phase and −84° in water) and one imaginary frequency of 690i cm^–1^ in the gas phase and 799i cm^–1^ in water (Scheme 11, Figure S11).

**Scheme 12:**
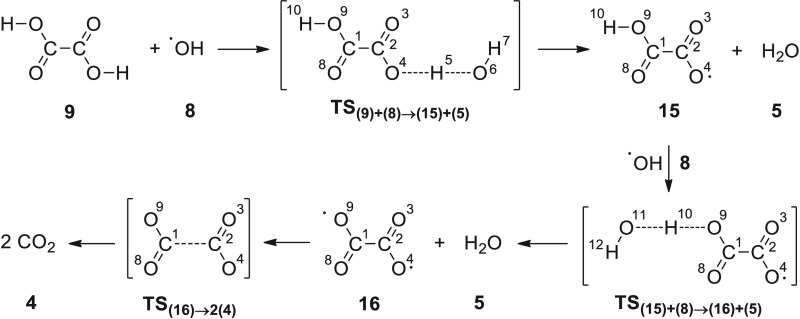
Reaction of Oxalic Acid with OH Radical

**Figure 4:**
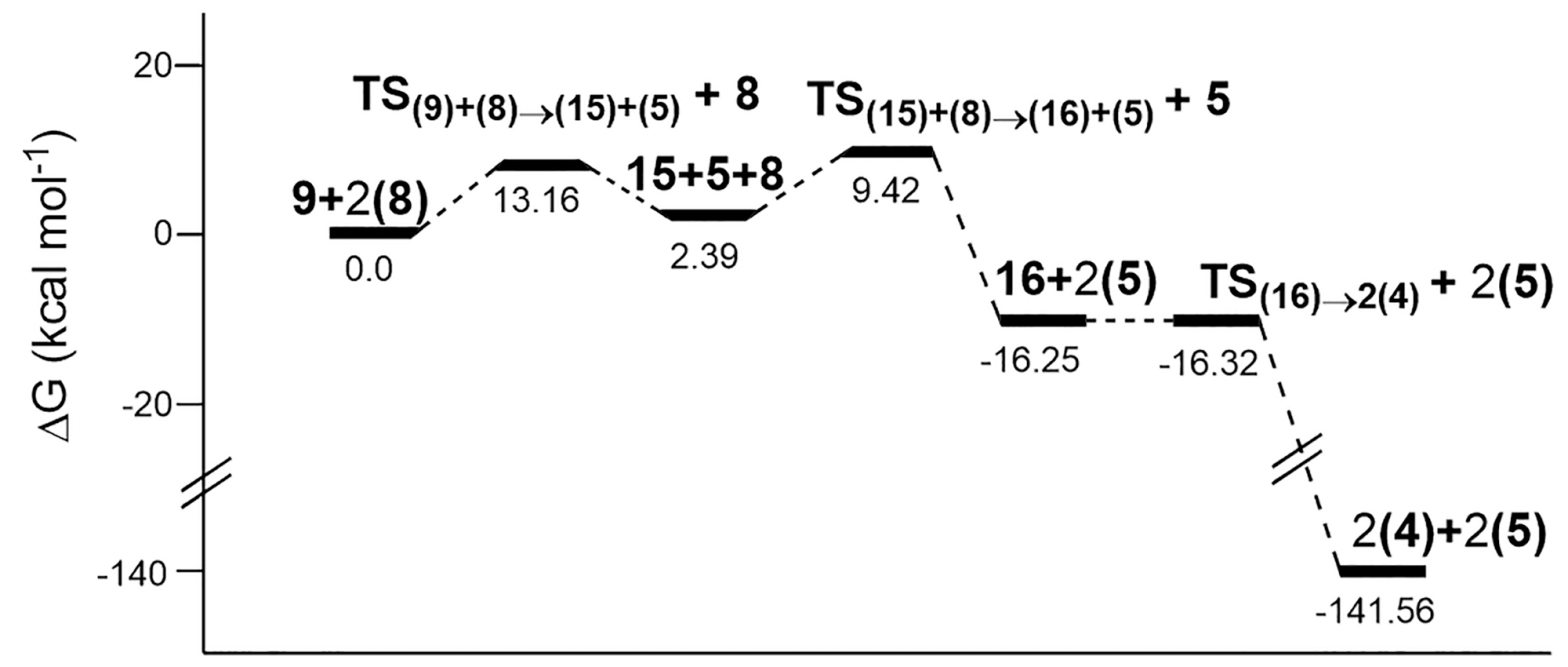
Relative free energies (Δ*G*, kcal mol^–1^) of all stationary points through the reaction of oxalic acid with OH radical, at the UMN12SX/6-311++G(2d,p) level with solvation in water (SMD method).

Finally, the H-atom abstraction in oxalic acid (**9**) by the OH radical (**8**) follows a reactivity similar to that observed for formic acid (Scheme 12), through a fast and concerted process leading to **TS**_**(9)+(8)→(15)+(5)**_ (Δ*G*^‡^ = 7.60 kcal mol^–1^ in the gas phase and 13.16 kcal mol^–1^ in water, with imaginary frequencies of 1748i and 1754i cm^–1^, respectively; Figures 4, S12 and S18), which enables the subsequent formation of radical **15**. Optimized structures are scarcely modified with respect to the attack of either ozone or OH radical to the carboxylate residue. Thus, calculated bond lengths for O^4^–H^5^ (1.20 Å in both media) and H^5^–O^6^ (1.21 Å in both media), along with identical O^4^–H^5^–O^6^ angles (156° in both media) and dihedral angles O^4^–H^5^–O^6^–H^7^ (85° in the gas phase and 88° in water), arise from the computational analysis.

The collision of H^10^ with a new hydroxyl radical (**8**) produces a diradical species (**16**) through the saddle point **TS**_**(15)+(8)→(16)+(5)**_ [bond distances O^11^–H^10^ (1.20 Å in the gas phase and 1.17 Å in water) and H^10^–O^9^ (1.21 Å in the gas phase and 1.24 Å in water), angle O^11^–H^10^–O^9^ (155° in both media), dihedral angle H^12^–O^11^–H^10^–O^9^ (83° in the gas phase and 86° in water), and imaginary frequencies 1774i cm^–1^ in the gas phase and 1663i cm^–1^ in water, Figure S13]. Conversion of **16** into two molecules of CO_2_ takes place via saddle point **TS**_**(16)→2(4)**_ [bond distances C^1^–C^2^ (1.91 Å in the gas phase and 1.88 Å in water), angles C^1^–C^2^–O^3^ (104° in the gas phase and 109° in water), dihedral angle O^8^–C^1^–C^2^–O^3^ (180° in both media), and imaginary frequencies 346i cm^–1^ in the gas phase and 279i cm^–1^ in water, Figure S14].

At this stage and in the context of hydrotrioxide chemistry,^42^ one could conjecture the intermediacy of such species by combining peroxy acid radicals and OH radicals because this pathway should likewise be plausible in the atmosphere. Nonetheless, as shown above, H-abstraction from organic acids should be the primary mechanism as formation of highly reactive hydroxyacyl and oxyacyl radicals is followed by essentially barrierless evolution to CO_2_ and H_2_O. It is obvious, however, that the present analysis focuses on ozonation and OH-based oxidation processes without taking into account the suite of different conditions, especially radiative or photochemical, which would permit secondary reaction channels.^48^ In line with experimental data gathered so far for gas-phase and aquatic reactions, H-abstraction mechanisms represent the main degradation routes of formic and oxalic acids, with competitive C–H and O–H cleavages depending on the parent substrate and the oxidizing agent as collected in Table 1.

**Table 1:** Comparative Reactivity (ΔΔ*G*^‡^, kcal/mol)^a^ of Formic and Oxalic Acids *versus* O_3_ and ^•^OH

Reaction step	Gas phase^b^	Water^b^
Cleavage of the C–H bond in HCOOH	15.11	14.65
Cleavage of the O–H bond in HCOOH	27.01	26.15
Cleavage of the O–H bond in HOOC–COOH	42.23	31.80

a Calculated as Δ*G*^‡^(O_3_) – Δ*G*^‡^(^•^OH) at the UMN12SX/6-311++G(2d,p) level of theory.

b Calculated from the reaction pathways involving the highest transition structures.

## Conclusions

In conclusion, this theoretical study using an open-shell DFT method, throws light on the reactivity of formic and oxalic acids with ozone and OH radicals. Such oxidative processes result from anthropogenic activity (e.g., destruction of pollutants by AOPs) and have therefore environmental importance as these low-carbon organic acids are usually the final products of chemical degradation. From a mechanistic standpoint, the interaction with ozone involves the formation of linear and highly oxygenated structures (hydrotrioxides). The computer-aided exploration suggests the feasible generation of these otherwise counterintuitive polyoxygenated species through H-abstraction and insertion reactions, whose geometries and energies have been thoroughly characterized. While hydrotrioxides have long been known in aquatic chemistry, their intermediacy in atmospheric chemistry has been evidenced very recently, albeit involving the formation of peroxy radicals prior to reaction with OH radicals, and having lifetimes from hours to days.^42^ In general, our computational study reveals higher reactivities for both acids when oxidized by OH radicals. The relative reactivity of formic acid against O_3_ and HO^•^ acid is essentially the same, regardless of the gas phase or in bulk aqueous solution (ΔΔ*G*^‡^ = 0.46 and 0.86 kcal mol^–1^, respectively). As expected, C–H bond oxidation is more favorable than O–H bond cleavage for formic acid. Large variations emerge when one compares the thermodynamic data of OH bond cleavage in oxalic acid, for which the oxidation process is more favored in water.

## Supporting Information Available

IRC analyses for saddle points (Figures S1–S14), thermochemistry data in the gas phase (Figures S15–S18), and computational data for all optimized structures at the UMN12SX/6-311++G(2d,p) level in the gas phase and water using the SMD method (PDF)
