# Supplementary material for: Mechanistic Insights into the Oxidative Degradation of Formic and Oxalic Acids with Ozone and OH Radical. A Computational Rationale
Source: J Phys Chem A. 2023 Feb 7;127(6):1491–8. doi: 10.1021/acs.jpca.2c08091 (PMC9940222; doi:10.1021/acs.jpca.2c08091)
Supplement: Supplementary file 1 — jp2c08091_si_001.pdf [file jp2c08091_si_001.pdf]

Supporting Information

Mechanistic Insights into the Oxidative Degradation of Formic and Oxalic Acids with Ozone and OH Radical. A Computational Rationale

Fernando J. Beltrán,[a,c,*] Ana María Chávez,[a,c] Pedro Cintas,[b,c] and R. Fernando Martínez[b,c,*]

[a]Departamento de Ingeniería Química y Química Física, Universidad de Extremadura, 06006 Badajoz, Spain

[b]Departamento de Química Orgánica e Inorgánica, Universidad de Extremadura, 06006 Badajoz, Spain

[c]Instituto del Agua, Cambio Climático y Sostenibilidad (IACYS), Universidad de Extremadura, 06006 Badajoz, Spain.

*Corresponding authors.

E-mail address: fbeltran@unex.es

E-mail address: rmarvaz@unex.es

Page	Supplementary Data
S2	Figures S1-S14. IRC analyses for saddle points
S9	Figures S15-S18. Thermochemistry data in gas phase
S11	Computational data for optimized structures at the UMN12SX/6-311++G(2d,p) level in water (SMD method)
S21	Computational data for optimized structures at the UMN12SX/6-311++G(2d,p) level in gas phase

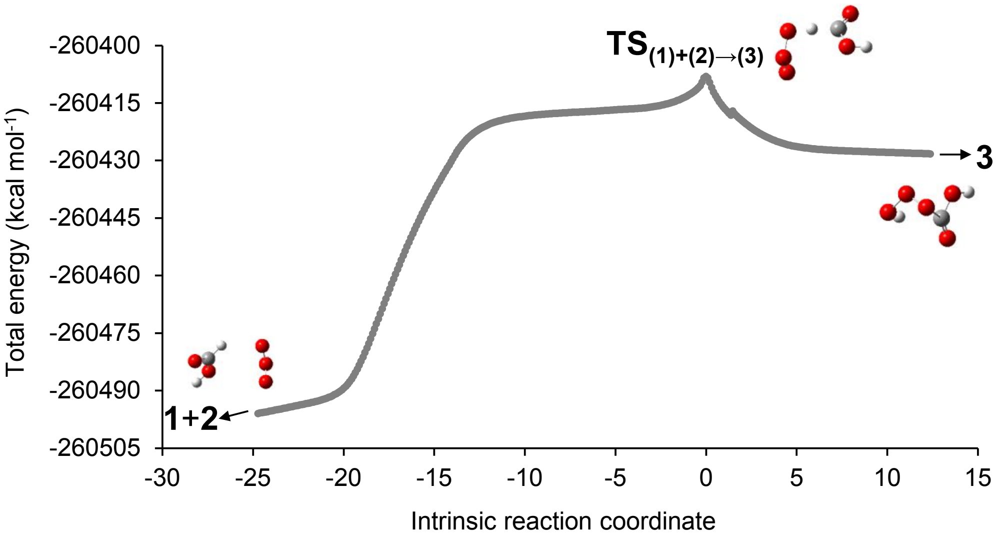

Figure S1. IRC analysis for transition state $\mathbf{TS}_{(1)+(2)\rightarrow(3)}$ at the UMN12SX/6-311++G(2d,p) level of theory as modeled with bulk solvation in water (SMD method).

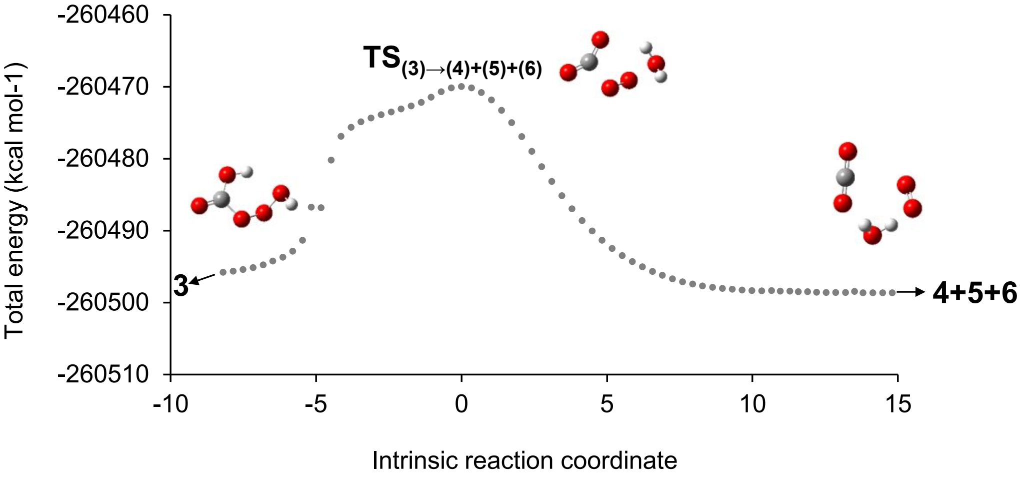

Figure S2. IRC analysis for transition state $\mathbf{TS}_{(3)\rightarrow(4)+(5)+(6)}$ at the UMN12SX/6-311++G(2d,p) level with bulk solvation in water (SMD method).

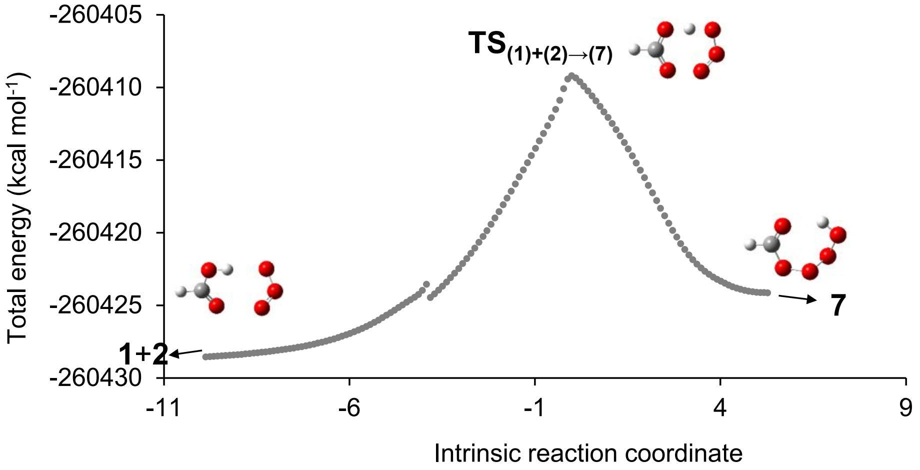

Figure S3. IRC analysis for transition state $\mathbf{TS}_{(1)+(2)\rightarrow(7)}$ at the UMN12SX/6-311++G(2d,p) level of theory including bulk solvation in water (SMD method).

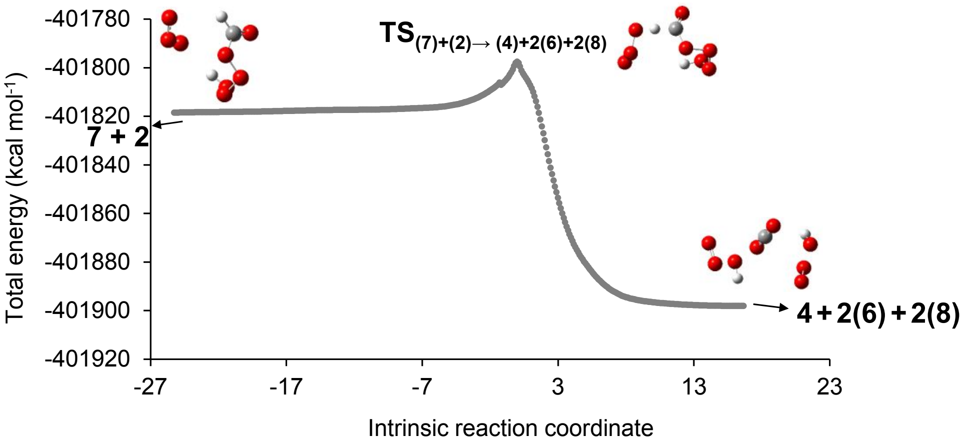

Figure S4. IRC analysis for transition state $\mathbf{TS}_{(7)+(2)\rightarrow(4)+2(6)+2(8)}$ at the UMN12SX/6-311++G(2d,p) level of theory with bulk solvation in water (SMD method).

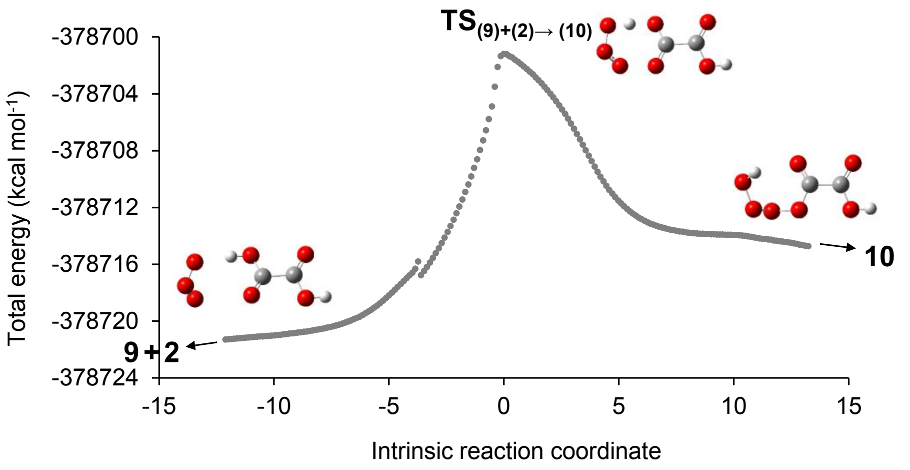

Figure S5. IRC analysis for transition state $\mathbf{TS}_{(9)+(2)\rightarrow(10)}$ at the UMN12SX/6-311++G(2d,p) level plus bulk solvation in water (SMD method).

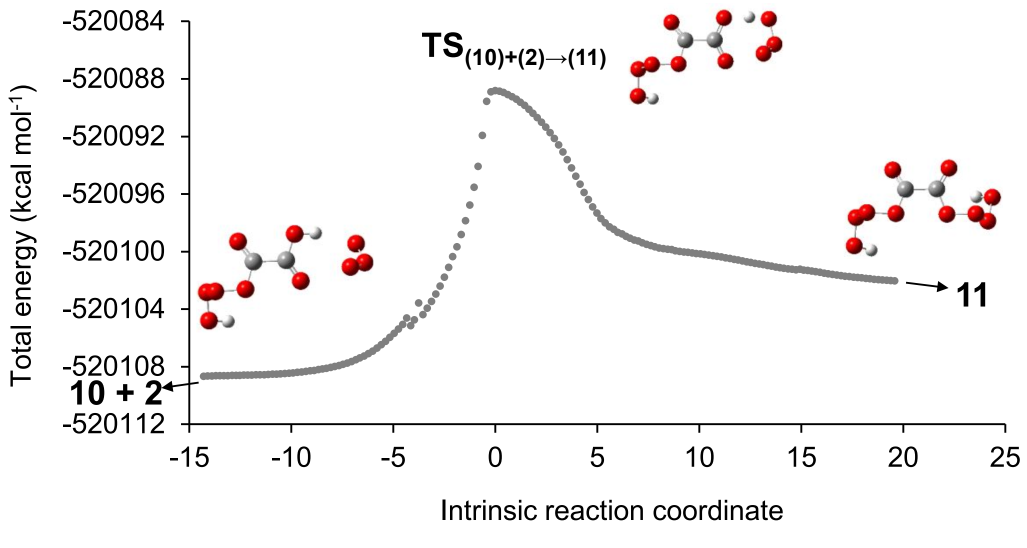

Figure S6. IRC analysis for transition state $\mathbf{TS}_{(10)+(2)\rightarrow(11)}$ at the UMN12SX/6-311++G(2d,p) level of theory as modeled with bulk solvation in water (SMD method).

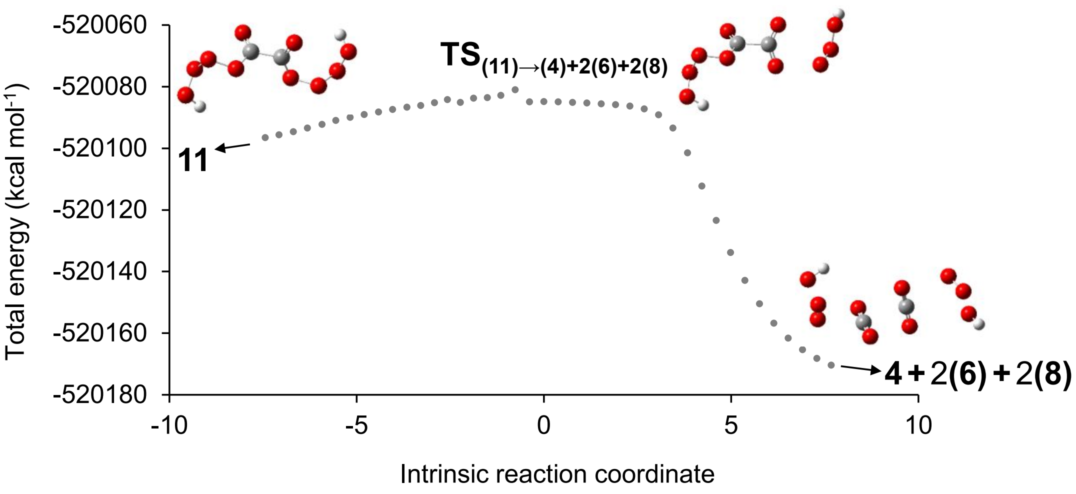

Figure S7. IRC analysis for transition state $\mathbf{TS}_{(11)\rightarrow(4)+2(6)+2(8)}$ at the UMN12SX/6-311++G(2d,p) level of theory as modeled with bulk solvation in water (SMD method).

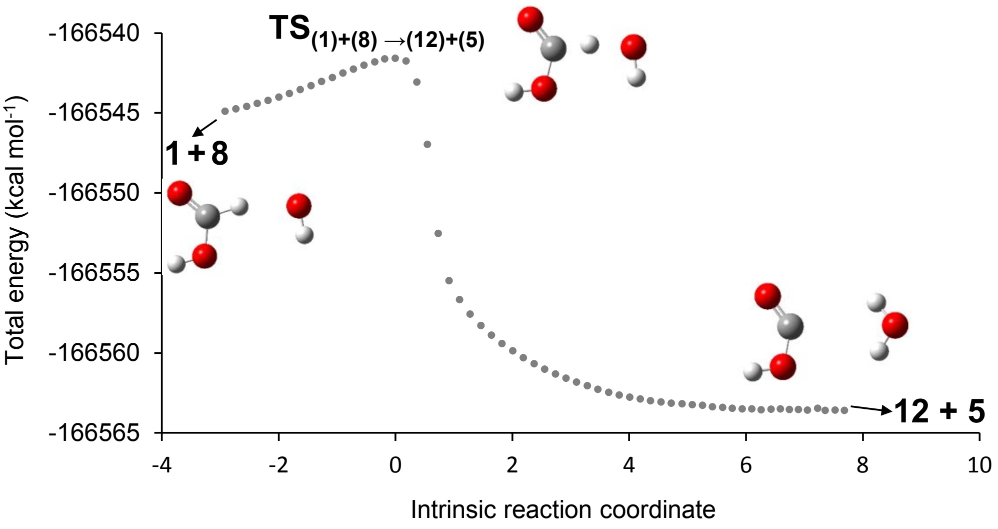

Figure S8. IRC analysis for transition state for $\mathbf{TS}_{(1)+(8)\rightarrow(12)+(5)}$ at the UMN12SX/6-311++G(2d,p) level of theory as modeled with bulk solvation in water (SMD method).

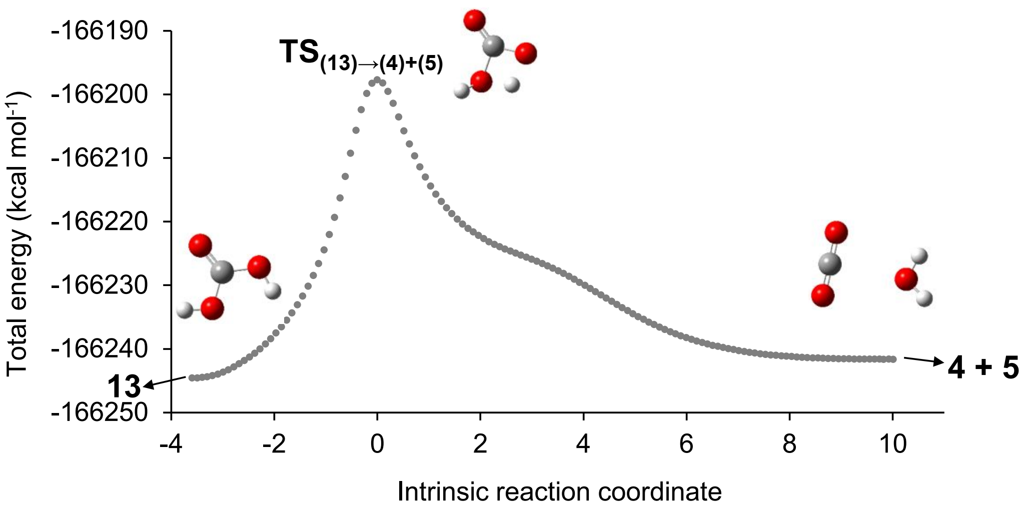

Figure S9. IRC analysis for transition state for $\mathbf{TS}_{(13)\rightarrow(4)+(5)}$ at the UMN12SX/6-311++G(2d,p) level of theory as modeled with bulk solvation in water (SMD method).

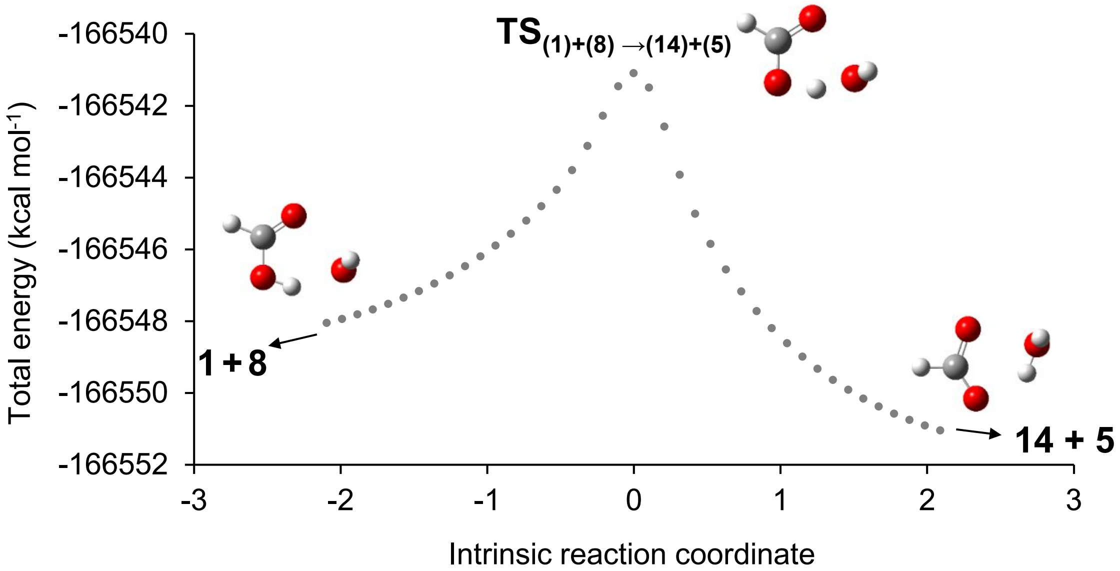

Figure S10. IRC analysis for transition state for $\mathbf{TS}_{(1)+(8)\rightarrow(14)+(5)}$ at the UMN12SX/6-311++G(2d,p) level of theory as modeled with bulk solvation in water (SMD method).

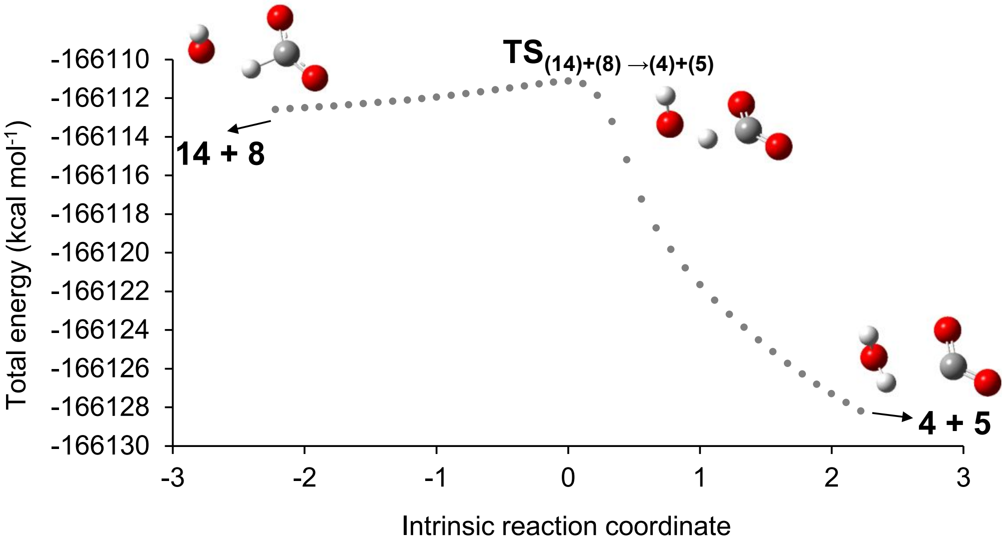

Figure S11. IRC analysis for transition state for $\mathbf{TS}_{(14)+(8)\rightarrow(4)+(5)}$ at the UMN12SX/6-311++G(2d,p) level of theory as modeled with bulk solvation in water (SMD method).

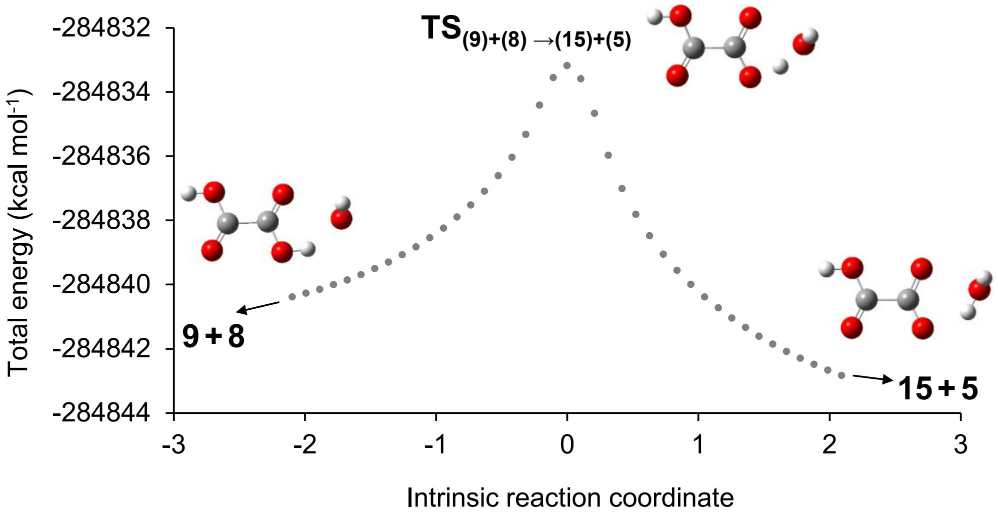

Figure S12. IRC analysis for transition state for $\mathbf{TS}_{(9)+(8)\rightarrow(15)+(5)}$ at the UMN12SX/6-311++G(2d,p) level of theory as modeled with bulk solvation in water (SMD method).

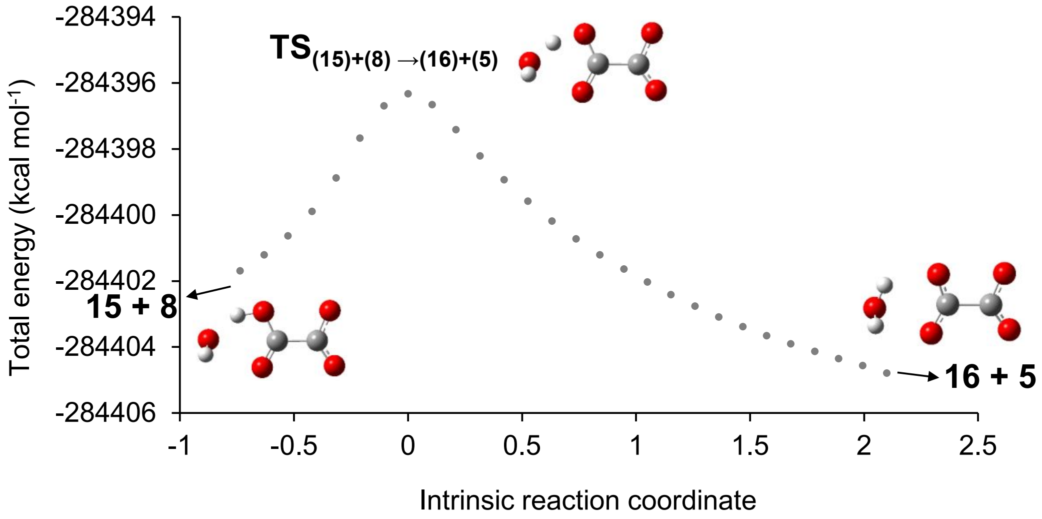

Figure S13. IRC analysis for transition state for $\mathbf{TS}_{(15)+(8)\rightarrow(16)+(5)}$ at the UMN12SX/6-311++G(2d,p) level of theory as modeled with bulk solvation in water (SMD method).

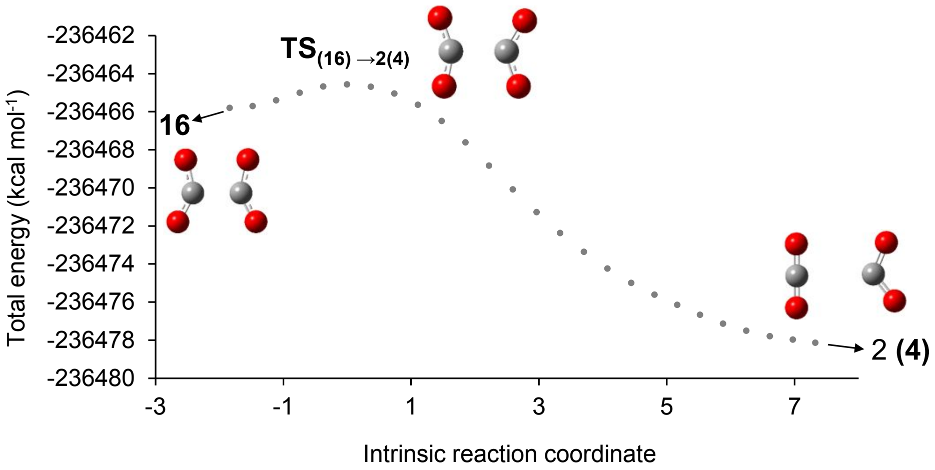

Figure S14. IRC analysis for transition state for $\mathbf{TS}_{(16)\rightarrow 2(4)}$ at the UMN12SX/6-311++G(2d,p) level of theory as modeled with bulk solvation in water (SMD method).

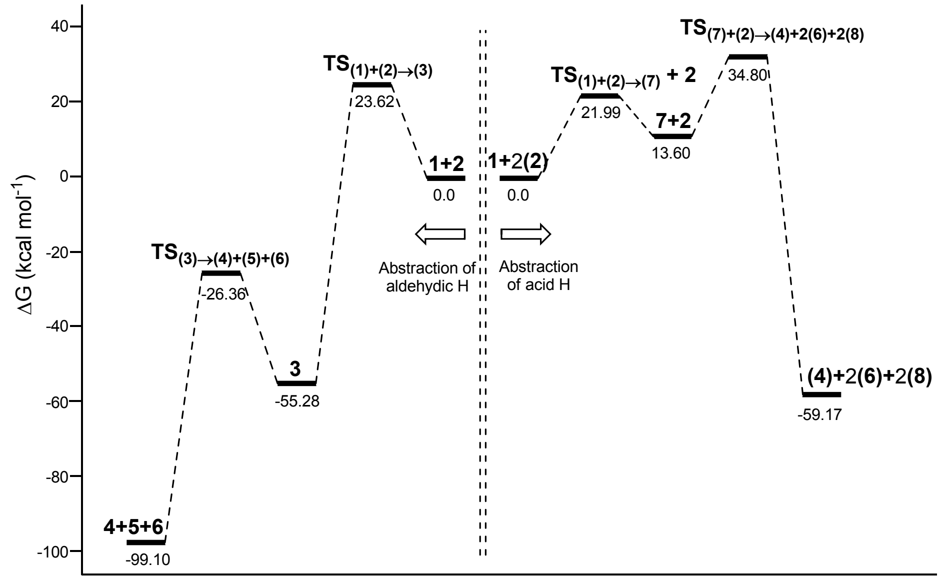

Figure S15. Relative free energies (ΔG, kcal mol^{-1}) of all stationary points involved in the reaction of formic acid with ozone, at the UMN12SX/6-311++G(2d,p) level of theory in gas phase.

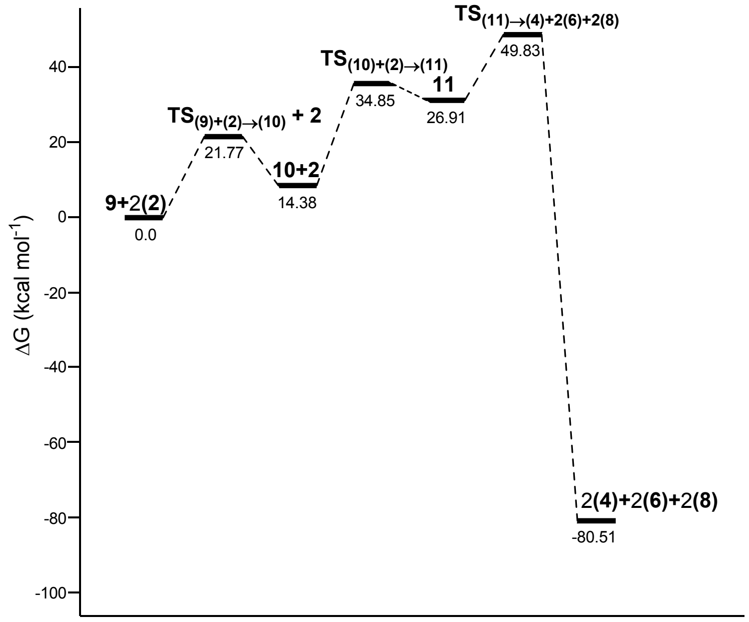

Figure S16. Relative free energies (ΔG, kcal mol^{-1}) for all stationary points involved in the reaction of oxalic acid with ozone, at the UMN12SX/6-311++G(2d,p) level in gas phase.

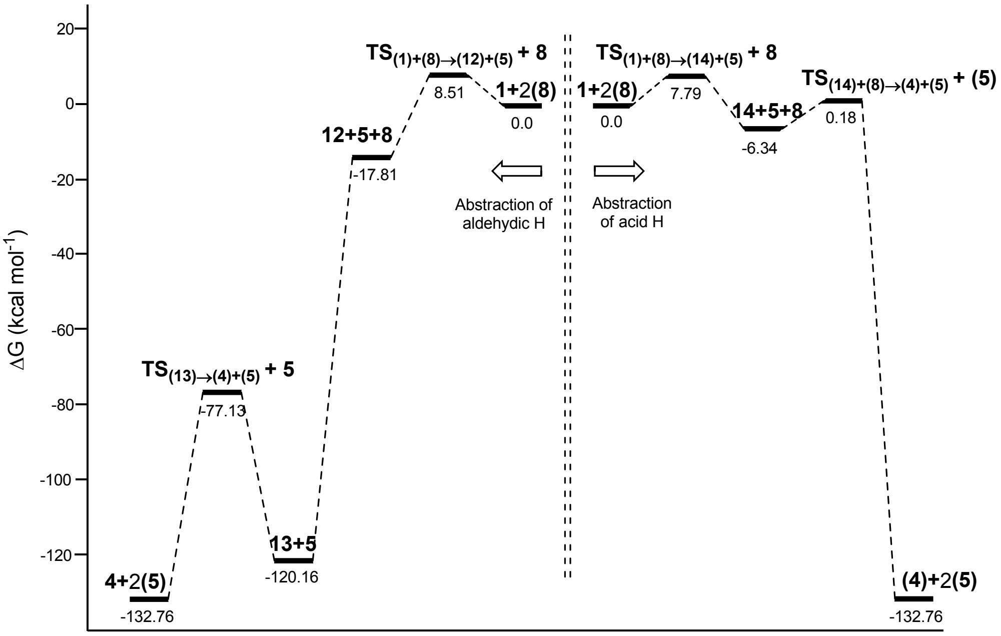

Figure S17. Relative free energy values (ΔG, kcal mol^{-1}) of all stationary points involved in the reaction of formic acid with OH radical, at the UMN12SX/6-311++G(2d,p) level in gas phase.

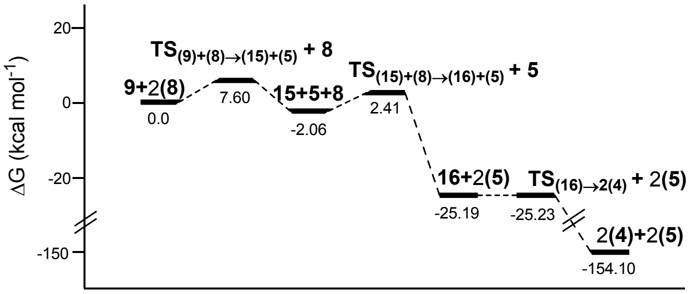

Figure S18. Relative free energies (ΔG, kcal mol^{-1}) of all stationary points through the reaction of oxalic acid with OH radical, at the UMN12SX/6-311++G(2d,p) level in gas phase.

Computational data for optimized structures at the UMN12SX/6-311++G(2d,p) level in water (SMD method)

Structure **1**

```
SCF Done:  E(UMN12SX) =  -189.696461047
Zero-point correction=                           0.033869 (Hartree/Particle)
Thermal correction to Energy=                    0.037063
Thermal correction to Enthalpy=                  0.038008
Thermal correction to Gibbs Free Energy=         0.009792
Sum of electronic and zero-point Energies=          -189.662592
Sum of electronic and thermal Energies=             -189.659398
Sum of electronic and thermal Enthalpies=           -189.658453
Sum of electronic and thermal Free Energies=        -189.686669

                        Standard orientation:
---------------------------------------------------------------------
Center     Atomic      Atomic             Coordinates (Angstroms)
Number     Number       Type             X           Y           Z
---------------------------------------------------------------------
     1          6           0        0.124617    0.400016    0.000176
     2          8           0        1.128009   -0.265267   -0.000086
     3          8           0       -1.101470   -0.089734   -0.000017
     4          1           0        0.110928    1.501937   -0.000180
     5          1           0       -1.070945   -1.062029   -0.000051
---------------------------------------------------------------------
```

Structure **2**

```
SCF Done:  E(UMN12SX) =  -225.319091561
Zero-point correction=                           0.007642 (Hartree/Particle)
Thermal correction to Energy=                    0.010587
Thermal correction to Enthalpy=                  0.011532
Thermal correction to Gibbs Free Energy=        -0.016086
Sum of electronic and zero-point Energies=          -225.311449
Sum of electronic and thermal Energies=             -225.308504
Sum of electronic and thermal Enthalpies=           -225.307560
Sum of electronic and thermal Free Energies=        -225.335178

                        Standard orientation:
---------------------------------------------------------------------
Center     Atomic      Atomic             Coordinates (Angstroms)
Number     Number       Type             X           Y           Z
---------------------------------------------------------------------
     1          8           0        0.000000    0.426358    0.000000
     2          8           0        1.062779   -0.213178    0.000000
     3          8           0       -1.062779   -0.213180    0.000000
---------------------------------------------------------------------
```

Structure $\mathbf{TS_{(1)+(2)\rightarrow(3)}}$

```
SCF Done:  E(UMN12SX) =  -414.986671001
Zero-point correction=                           0.036742 (Hartree/Particle)
Thermal correction to Energy=                    0.043485
Thermal correction to Enthalpy=                  0.044429
Thermal correction to Gibbs Free Energy=         0.004631
Sum of electronic and zero-point Energies=          -414.949929
Sum of electronic and thermal Energies=             -414.943186
Sum of electronic and thermal Enthalpies=           -414.942242
Sum of electronic and thermal Free Energies=        -414.982040

One imaginary frequency: -1873.3716i cm-1

                        Standard orientation:
---------------------------------------------------------------------
Center     Atomic      Atomic             Coordinates (Angstroms)
Number     Number       Type             X           Y           Z
---------------------------------------------------------------------
     1          6           0        1.314860   -0.109899   -0.000014
     2          8           0        2.158704   -0.784172    0.490300
     3          8           0        1.324492    1.132635   -0.374106
     4          1           0        0.103844   -0.609092   -0.269948
     5          1           0        2.195004    1.546799   -0.211648
     6          8           0       -1.008930   -0.939521   -0.573105
     7          8           0       -1.784774    0.160178   -0.361579
     8          8           0       -1.962993    0.396090    0.878700
---------------------------------------------------------------------
```

Structure **3**

```
SCF Done:  E(UMN12SX) =  -415.130342
Zero-point correction=                           0.046182 (Hartree/Particle)
Thermal correction to Energy=                    0.052089
Thermal correction to Enthalpy=                  0.053033
Thermal correction to Gibbs Free Energy=         0.016416
Sum of electronic and zero-point Energies=           -415.084160
Sum of electronic and thermal Energies=              -415.078253
Sum of electronic and thermal Enthalpies=            -415.077309
Sum of electronic and thermal Free Energies=         -415.113926
```

Standard orientation:

Center Number	Atomic Number	Atomic Type	Coordinates (Angstroms) X	Y	Z
1	1	0	2.503319	-0.753185	0.558236
2	1	0	0.012155	1.543179	-0.297134
3	8	0	1.970990	0.061299	0.596088
4	8	0	1.242335	0.011426	-0.590926
5	8	0	0.089820	-0.786342	-0.333420
6	8	0	-0.871250	1.261883	-0.001482
7	6	0	-0.995881	-0.033598	0.010238
8	8	0	-1.999419	-0.621817	0.289424

Structure $\mathbf{TS_{(3)\rightarrow(4)+(5)+(6)}}$

```
SCF Done:  E(UMN12SX) =  -415.085352
Zero-point correction=                           0.043799 (Hartree/Particle)
Thermal correction to Energy=                    0.049926
Thermal correction to Enthalpy=                  0.050870
Thermal correction to Gibbs Free Energy=         0.013670
Sum of electronic and zero-point Energies=           -415.041553
Sum of electronic and thermal Energies=              -415.035426
Sum of electronic and thermal Enthalpies=            -415.034482
Sum of electronic and thermal Free Energies=         -415.071682
```

One imaginary frequency: -382.13i cm-1

Standard orientation:

Center Number	Atomic Number	Atomic Type	Coordinates (Angstroms) X	Y	Z
1	1	0	2.121888	-0.061020	1.193671
2	1	0	1.454811	1.141709	0.410699
3	8	0	2.064292	0.378516	0.321535
4	8	0	1.073537	-0.644396	-0.579167
5	8	0	0.019650	-0.961608	0.075416
6	8	0	-0.611179	1.252181	-0.303681
7	6	0	-1.116197	0.203405	-0.006512
8	8	0	-2.156239	-0.312332	0.290234

Structure **4**

```
SCF Done:  E(UMN12SX) =  -188.511376
Zero-point correction=                           0.011707 (Hartree/Particle)
Thermal correction to Energy=                    0.014321
Thermal correction to Enthalpy=                  0.015266
Thermal correction to Gibbs Free Energy=        -0.008972
Sum of electronic and zero-point Energies=           -188.499669
Sum of electronic and thermal Energies=              -188.497054
Sum of electronic and thermal Enthalpies=            -188.496110
Sum of electronic and thermal Free Energies=         -188.520347
```

Standard orientation:

Center Number	Atomic Number	Atomic Type	Coordinates (Angstroms) X	Y	Z
1	6	0	0.000000	0.000000	0.000000
2	8	0	0.000000	0.000000	1.153132
3	8	0	0.000000	0.000000	-1.153132

Structure **5**

```
 SCF Done:  E(UMN12SX) =  -76.4089911433
 Zero-point correction=                           0.021482 (Hartree/Particle)
 Thermal correction to Energy=                    0.024318
 Thermal correction to Enthalpy=                  0.025262
 Thermal correction to Gibbs Free Energy=         0.003831
 Sum of electronic and zero-point Energies=           -76.387509
 Sum of electronic and thermal Energies=              -76.384674
 Sum of electronic and thermal Enthalpies=            -76.383729
 Sum of electronic and thermal Free Energies=         -76.405160

                        Standard orientation:
 ---------------------------------------------------------------------
 Center     Atomic      Atomic             Coordinates (Angstroms)
 Number     Number       Type             X           Y           Z
 ---------------------------------------------------------------------
      1          8           0        0.000000    0.000000    0.117838
      2          1           0       -0.000000    0.763602   -0.471351
      3          1           0       -0.000000   -0.763602   -0.471351
 ---------------------------------------------------------------------
```

Structure **6**

```
 SCF Done:  E(UMN12SX) = -150.250079
 Zero-point correction=                           0.003884 (Hartree/Particle)
 Thermal correction to Energy=                    0.006246
 Thermal correction to Enthalpy=                  0.007191
 Thermal correction to Gibbs Free Energy=        -0.016069
 Sum of electronic and zero-point Energies=          -150.246195
 Sum of electronic and thermal Energies=             -150.243832
 Sum of electronic and thermal Enthalpies=           -150.242888
 Sum of electronic and thermal Free Energies=        -150.266148

                        Standard orientation:
 ---------------------------------------------------------------------
 Center     Atomic      Atomic             Coordinates (Angstroms)
 Number     Number       Type             X           Y           Z
 ---------------------------------------------------------------------
      1          8           0        0.000000    0.000000    0.598989
      2          8           0        0.000000    0.000000   -0.598989
 ---------------------------------------------------------------------
```

Structure $\mathbf{TS_{(1)+(2)\rightarrow(7)}}$

```
 SCF Done:  E(UMN12SX) =  -414.988458
 Zero-point correction=                           0.039174 (Hartree/Particle)
 Thermal correction to Energy=                    0.044734
 Thermal correction to Enthalpy=                  0.045679
 Thermal correction to Gibbs Free Energy=         0.009724
 Sum of electronic and zero-point Energies=          -414.949284
 Sum of electronic and thermal Energies=             -414.943723
 Sum of electronic and thermal Enthalpies=           -414.942779
 Sum of electronic and thermal Free Energies=        -414.978734

One imaginary frequency: -1232.25i cm-1

                        Standard orientation:
 ---------------------------------------------------------------------
 Center     Atomic      Atomic             Coordinates (Angstroms)
 Number     Number       Type             X           Y           Z
 ---------------------------------------------------------------------
      1          1           0        0.239254    1.135449    0.099001
      2          1           0       -2.691873    0.133213   -0.134319
      3          8           0        1.390688    0.996486   -0.045090
      4          8           0        1.463578   -0.345338   -0.299073
      5          8           0        0.722497   -1.008954    0.439804
      6          8           0       -0.991983    1.172457    0.147548
      7          6           0       -1.588785    0.092955   -0.056867
      8          8           0       -1.086613   -1.042950   -0.196125
 ---------------------------------------------------------------------
```

Structure **7**

```
 SCF Done:  E(UMN12SX) =  -415.014714943
 Zero-point correction=                           0.044846 (Hartree/Particle)
 Thermal correction to Energy=                    0.050814
 Thermal correction to Enthalpy=                  0.051759
 Thermal correction to Gibbs Free Energy=         0.014882
 Sum of electronic and zero-point Energies=          -414.969869
 Sum of electronic and thermal Energies=             -414.963901
 Sum of electronic and thermal Enthalpies=           -414.962956
 Sum of electronic and thermal Free Energies=        -414.999833
```

Standard orientation:

Center Number	Atomic Number	Atomic Type	X	Y	Z
1	1	0	1.166413	1.128659	-1.065043
2	1	0	-2.300144	-0.234386	-1.096633
3	8	0	1.550237	1.016893	-0.176867
4	8	0	1.553626	-0.378455	-0.016181
5	8	0	0.396884	-0.744246	0.634168
6	8	0	-1.592575	0.924650	0.444690
7	6	0	-1.558845	0.011548	-0.316314
8	8	0	-0.597321	-0.939287	-0.378366

Structure **TS$_{(7)+(2)\rightarrow(4)+2(6)+2(8)}$**

```
SCF Done:  E(UMN12SX) =  -640.305104
Zero-point correction=                           0.047061 (Hartree/Particle)
Thermal correction to Energy=                    0.056973
Thermal correction to Enthalpy=                  0.057917
Thermal correction to Gibbs Free Energy=         0.010020
Sum of electronic and zero-point Energies=           -640.258043
Sum of electronic and thermal Energies=              -640.248131
Sum of electronic and thermal Enthalpies=            -640.247187
Sum of electronic and thermal Free Energies=         -640.295084
```

One imaginary frequency: -2014.70i cm-1

Standard orientation:

Center Number	Atomic Number	Atomic Type	X	Y	Z
1	1	0	-1.610674	-1.276898	1.241789
2	1	0	1.350958	0.896286	0.036977
3	8	0	-2.462011	-0.885677	0.973072
4	8	0	-2.374878	-0.909696	-0.431029
5	8	0	-1.941692	0.294188	-0.850754
6	8	0	-0.490966	1.797827	0.839429
7	8	0	-0.440659	0.235133	-0.808288
8	8	0	2.546429	0.710193	-0.024246
9	8	0	2.702397	-0.572400	-0.427768
10	8	0	2.468532	-1.409781	0.500416
11	6	0	0.033752	1.050384	0.092429

Structure **8**

```
SCF Done:  E(UMN12SX) =  -75.7104009713
Zero-point correction=                           0.008597 (Hartree/Particle)
Thermal correction to Energy=                    0.010958
Thermal correction to Enthalpy=                  0.011902
Thermal correction to Gibbs Free Energy=        -0.008334
Sum of electronic and zero-point Energies=            -75.701804
Sum of electronic and thermal Energies=               -75.699443
Sum of electronic and thermal Enthalpies=             -75.698499
Sum of electronic and thermal Free Energies=          -75.718735
```

Standard orientation:

Center Number	Atomic Number	Atomic Type	X	Y	Z
1	8	0	0.000000	0.000000	0.108613
2	1	0	0.000000	0.000000	-0.868907

Structure **9**

```
SCF Done:  E(UMN12SX) = -378.207359
Zero-point correction=                           0.049255 (Hartree/Particle)
Thermal correction to Energy=                    0.054815
Thermal correction to Enthalpy=                  0.055759
Thermal correction to Gibbs Free Energy=         0.019408
Sum of electronic and zero-point Energies=           -378.158104
Sum of electronic and thermal Energies=              -378.152544
Sum of electronic and thermal Enthalpies=            -378.151600
Sum of electronic and thermal Free Energies=         -378.187951
```

Standard orientation:

Center Number	Atomic Number	Atomic Type	Coordinates (Angstroms) X	Y	Z
1	1	0	2.409513	-0.729936	-0.000030
2	8	0	1.460817	-0.944457	-0.000027
3	6	0	0.752062	0.158582	0.000002
4	8	0	1.180042	1.279682	0.000028
5	6	0	-0.752063	-0.158582	0.000004
6	8	0	-1.180042	-1.279683	0.000028
7	8	0	-1.460817	0.944456	-0.000025
8	1	0	-2.409513	0.729940	-0.000044

Structure $\mathbf{TS}_{\mathbf{(9)+(2)\rightarrow(10)}}$

```
SCF Done:  E(UMN12SX) =  -603.498769
Zero-point correction=                           0.054930 (Hartree/Particle)
Thermal correction to Energy=                    0.063223
Thermal correction to Enthalpy=                  0.064167
Thermal correction to Gibbs Free Energy=         0.020517
Sum of electronic and zero-point Energies=           -603.443839
Sum of electronic and thermal Energies=              -603.435547
Sum of electronic and thermal Enthalpies=            -603.434602
Sum of electronic and thermal Free Energies=         -603.478252
```

One imaginary frequency: -721.99i cm-1

Standard orientation:

Center Number	Atomic Number	Atomic Type	Coordinates (Angstroms) X	Y	Z
1	1	0	1.419499	1.113576	0.120604
2	8	0	2.507289	1.016220	-0.122941
3	8	0	2.653039	-0.325953	-0.284730
4	8	0	1.932931	-0.987760	0.452126
5	8	0	0.120166	1.117932	0.248163
6	6	0	-0.458368	0.043567	-0.000056
7	8	0	0.041202	-1.065446	-0.247323
8	6	0	-2.009652	0.122351	-0.017162
9	8	0	-2.582878	1.172771	-0.057176
10	8	0	-2.557271	-1.067849	0.009700
11	1	0	-3.527200	-0.988398	0.000154

Structure **10**

```
SCF Done:  E(UMN12SX) = -603.522029
Zero-point correction=                           0.060206 (Hartree/Particle)
Thermal correction to Energy=                    0.068845
Thermal correction to Enthalpy=                  0.069789
Thermal correction to Gibbs Free Energy=         0.025257
Sum of electronic and zero-point Energies=           -603.461823
Sum of electronic and thermal Energies=              -603.453184
Sum of electronic and thermal Enthalpies=            -603.452240
Sum of electronic and thermal Free Energies=         -603.496772
```

Standard orientation:

Center Number	Atomic Number	Atomic Type	Coordinates (Angstroms) X	Y	Z
1	1	0	1.846705	0.369646	-1.645822
2	8	0	2.507373	0.569158	-0.958573
3	8	0	2.473717	-0.580903	-0.154096
4	8	0	1.611217	-0.350581	0.887969
5	8	0	-0.183554	1.491260	0.538190
6	6	0	-0.485288	0.365076	0.316182
7	8	0	0.302657	-0.718346	0.419585
8	6	0	-1.904329	-0.035458	-0.135029
9	8	0	-2.730768	0.813572	-0.300036
10	8	0	-2.049156	-1.325934	-0.292558
11	1	0	-2.960883	-1.533150	-0.564944

Structure $\mathbf{TS}_{\mathbf{(10)+(2)\rightarrow(11)}}$

```
SCF Done:  E(UMN12SX) =  -828.814276
Zero-point correction=                           0.066175 (Hartree/Particle)
Thermal correction to Energy=                    0.077780
Thermal correction to Enthalpy=                  0.078725
Thermal correction to Gibbs Free Energy=         0.026251
Sum of electronic and zero-point Energies=           -828.748102
Sum of electronic and thermal Energies=              -828.736496
Sum of electronic and thermal Enthalpies=            -828.735552
```

```
 Sum of electronic and thermal Free Energies=         -828.788026
```

One imaginary frequency: -336.54i cm-1

Standard orientation:

Center Number	Atomic Number	Atomic Type	Coordinates (Angstroms) X	Y	Z
1	1	0	2.842904	0.873315	-0.272178
2	8	0	3.815723	0.462006	-0.540027
3	8	0	3.677248	-0.861514	-0.266548
4	8	0	2.900445	-1.097223	0.642180
5	8	0	1.548530	1.245028	-0.054597
6	6	0	0.736198	0.312025	0.036386
7	8	0	0.942838	-0.911669	0.038655
8	6	0	-0.745903	0.760548	0.156867
9	8	0	-1.135509	1.849728	-0.101151
10	8	0	-1.490787	-0.275546	0.587328
11	8	0	-2.845335	0.132097	0.780110
12	8	0	-3.476845	0.041133	-0.443923
13	8	0	-3.891456	-1.287044	-0.615941
14	1	0	-3.143480	-1.684715	-1.096040

Structure **11**

```
 SCF Done:  E(UMN12SX) = -828.836025
 Zero-point correction=                           0.070085 (Hartree/Particle)
 Thermal correction to Energy=                    0.082157
 Thermal correction to Enthalpy=                  0.083102
 Thermal correction to Gibbs Free Energy=         0.029841
 Sum of electronic and zero-point Energies=           -828.765941
 Sum of electronic and thermal Energies=              -828.753868
 Sum of electronic and thermal Enthalpies=            -828.752924
 Sum of electronic and thermal Free Energies=         -828.806184
```

Standard orientation:

Center Number	Atomic Number	Atomic Type	Coordinates (Angstroms) X	Y	Z
1	1	0	2.280406	-1.258277	-1.501676
2	8	0	3.156349	-1.014187	-1.151389
3	8	0	3.030513	-1.298099	0.217841
4	8	0	2.659367	-0.148349	0.865645
5	8	0	1.503101	1.762869	-0.472490
6	6	0	0.825049	0.957122	0.070122
7	8	0	1.219987	-0.094851	0.797466
8	6	0	-0.717286	1.030303	0.002735
9	8	0	-1.268714	1.885261	-0.603235
10	8	0	-1.279422	0.024214	0.687030
11	8	0	-2.708039	0.131239	0.652081
12	8	0	-3.133195	-0.466164	-0.515050
13	8	0	-3.248876	-1.842414	-0.277094
14	1	0	-2.375566	-2.182428	-0.541905

Structure **TS**$_{(11)\rightarrow(4)+2(6)+2(8)}$

```
 SCF Done:  E(UMN12SX) =  -828.807941
 Zero-point correction=                           0.064511 (Hartree/Particle)
 Thermal correction to Energy=                    0.078059
 Thermal correction to Enthalpy=                  0.079003
 Thermal correction to Gibbs Free Energy=         0.021277
 Sum of electronic and zero-point Energies=           -828.743430
 Sum of electronic and thermal Energies=              -828.729882
 Sum of electronic and thermal Enthalpies=            -828.728938
 Sum of electronic and thermal Free Energies=         -828.786664
```

One imaginary frequency: -214.82i cm-1

Standard orientation:

Center Number	Atomic Number	Atomic Type	Coordinates (Angstroms) X	Y	Z
1	1	0	4.675323	-1.003360	0.540074
2	8	0	4.271126	-0.488573	-0.183550
3	8	0	3.846247	0.671807	0.526875
4	8	0	3.063093	1.333793	-0.186069
5	8	0	1.342403	-1.149891	-0.699945
6	6	0	0.686371	-0.291495	-0.177414
7	8	0	0.818923	0.868533	0.228657
8	6	0	-0.846752	-0.711747	0.161882
9	8	0	-1.102401	-1.488533	1.009568
10	8	0	-1.641206	-0.077091	-0.686423
11	8	0	-3.008165	-0.549954	-0.540760

12	8	0	-3.574088	0.151181	0.487288
13	8	0	-4.063642	1.359210	-0.029050
14	1	0	-3.331357	1.978972	0.140402

Structure $\mathbf{TS}_{(1)+(8)\rightarrow(12)+(5)}$

```
SCF Done:  E(UMN12SX) =  -265.400888
Zero-point correction=                           0.041088 (Hartree/Particle)
Thermal correction to Energy=                    0.046346
Thermal correction to Enthalpy=                  0.047290
Thermal correction to Gibbs Free Energy=         0.011950
Sum of electronic and zero-point Energies=           -265.359800
Sum of electronic and thermal Energies=              -265.354542
Sum of electronic and thermal Enthalpies=            -265.353598
Sum of electronic and thermal Free Energies=         -265.388938
```

One imaginary frequency: -555.52i cm-1

Standard orientation:

Center Number	Atomic Number	Atomic Type	Coordinates (Angstroms) X	Y	Z
1	6	0	-0.448487	-0.137031	-0.000012
2	8	0	-1.188099	-1.074822	-0.000098
3	8	0	-0.768847	1.133059	0.000046
4	1	0	0.723927	-0.262919	0.000032
5	1	0	-1.738264	1.241950	0.000001
6	8	0	2.141761	-0.177046	0.000059
7	1	0	2.226736	0.793624	-0.000018

Structure **12**

```
SCF Done:  E(UMN12SX) =  -189.024574950
Zero-point correction=                           0.020766 (Hartree/Particle)
Thermal correction to Energy=                    0.023992
Thermal correction to Enthalpy=                  0.024936
Thermal correction to Gibbs Free Energy=        -0.003655
Sum of electronic and zero-point Energies=           -189.003809
Sum of electronic and thermal Energies=              -189.000583
Sum of electronic and thermal Enthalpies=            -188.999639
Sum of electronic and thermal Free Energies=         -189.028230
```

Standard orientation:

Center Number	Atomic Number	Atomic Type	Coordinates (Angstroms) X	Y	Z
1	6	0	0.122946	-0.412469	-0.000089
2	8	0	1.155105	0.170245	0.000011
3	8	0	-1.104241	0.014940	0.000046
4	1	0	-1.144590	0.993334	0.000074

Structure **13**

```
SCF Done:  E(UMN12SX) =  -264.927599
Zero-point correction=                           0.039643 (Hartree/Particle)
Thermal correction to Energy=                    0.043379
Thermal correction to Enthalpy=                  0.044324
Thermal correction to Gibbs Free Energy=         0.013756
Sum of electronic and zero-point Energies=           -264.887956
Sum of electronic and thermal Energies=              -264.884220
Sum of electronic and thermal Enthalpies=            -264.883275
Sum of electronic and thermal Free Energies=         -264.913843
```

Standard orientation:

Center Number	Atomic Number	Atomic Type	Coordinates (Angstroms) X	Y	Z
1	6	0	0.040928	-0.117190	-0.000751
2	8	0	0.611473	-1.179317	0.000313
3	8	0	-1.275057	-0.030521	0.000219
4	1	0	-1.566869	0.894132	0.000226
5	8	0	0.629717	1.067418	-0.000041
6	1	0	1.592239	0.948362	0.000355

Structure $\mathbf{TS}_{(13)\rightarrow(4)+(5)}$

```
SCF Done:  E(UMN12SX) =  -264.852941
Zero-point correction=                           0.034153 (Hartree/Particle)
Thermal correction to Energy=                    0.037691
Thermal correction to Enthalpy=                  0.038635
Thermal correction to Gibbs Free Energy=         0.008347
Sum of electronic and zero-point Energies=           -264.818788
Sum of electronic and thermal Energies=              -264.815250
Sum of electronic and thermal Enthalpies=            -264.814306
Sum of electronic and thermal Free Energies=         -264.844594
```

One imaginary frequency: -2024.71i cm-1

Standard orientation:

Center Number	Atomic Number	Atomic Type	X	Y	Z
1	1	0	0.995734	-1.442890	0.610650
2	1	0	1.334075	0.342110	0.047278
3	8	0	0.926157	-0.780905	-0.100893
4	8	0	0.336555	1.197417	0.025018
5	6	0	-0.248476	0.073091	-0.009024
6	8	0	-1.367581	-0.333733	0.000401

Coordinates (Angstroms)

Structure **TS$_{(1)+(8)\rightarrow(14)+(5)}$**

```
SCF Done:  E(UMN12SX) =  -265.400130584
Zero-point correction=                           0.042295 (Hartree/Particle)
Thermal correction to Energy=                    0.046646
Thermal correction to Enthalpy=                  0.047591
Thermal correction to Gibbs Free Energy=         0.015005
Sum of electronic and zero-point Energies=           -265.357836
Sum of electronic and thermal Energies=              -265.353484
Sum of electronic and thermal Enthalpies=            -265.352540
Sum of electronic and thermal Free Energies=         -265.385126
```

One imaginary frequency: -1747.34i cm-1

Standard orientation:

Center Number	Atomic Number	Atomic Type	X	Y	Z
1	1	0	0.611122	-0.805956	-0.016672
2	1	0	-2.112420	0.232474	0.041598
3	8	0	1.578986	-0.075078	-0.099093
4	8	0	-0.551791	-1.073663	0.011288
5	6	0	-1.016330	0.098177	0.010005
6	8	0	-0.299054	1.114549	-0.022014
7	1	0	1.774144	0.257959	0.793603

Coordinates (Angstroms)

Structure **14 + 5**

```
SCF Done:  E(UMN12SX) =  -265.416964576
Zero-point correction=                           0.043987 (Hartree/Particle)
Thermal correction to Energy=                    0.050249
Thermal correction to Enthalpy=                  0.051193
Thermal correction to Gibbs Free Energy=         0.014208
Sum of electronic and zero-point Energies=           -265.372978
Sum of electronic and thermal Energies=              -265.366715
Sum of electronic and thermal Enthalpies=            -265.365771
Sum of electronic and thermal Free Energies=         -265.402757
```

Standard orientation:

Center Number	Atomic Number	Atomic Type	X	Y	Z
1	1	0	1.187429	-0.926576	-0.015505
2	1	0	-2.085727	0.643519	0.041871
3	8	0	1.789125	-0.170602	-0.097691
4	8	0	-0.909199	-1.036228	0.008686
5	6	0	-1.074331	0.160193	0.009590
6	8	0	-0.207361	1.108183	-0.022769
7	1	0	1.963764	0.111075	0.810286

Coordinates (Angstroms)

Structure **TS$_{(14)+(8)\rightarrow(4)+(5)}$**

```
SCF Done:  E(UMN12SX) =  -264.714894
Zero-point correction=                           0.027707 (Hartree/Particle)
Thermal correction to Energy=                    0.032479
Thermal correction to Enthalpy=                  0.033423
Thermal correction to Gibbs Free Energy=        -0.000565
Sum of electronic and zero-point Energies=           -264.687187
Sum of electronic and thermal Energies=              -264.682415
Sum of electronic and thermal Enthalpies=            -264.681471
Sum of electronic and thermal Free Energies=         -264.715459
```

One imaginary frequency: -798.62i cm-1

Standard orientation:

Center Number	Atomic Number	Atomic Type	Coordinates (Angstroms) X	Y	Z
1	6	0	0.568346	-0.035225	-0.010820
2	8	0	1.662194	-0.525754	0.019481
3	8	0	0.038888	1.084098	-0.008593
4	1	0	-0.515446	-0.759536	-0.060635
5	8	0	-1.809928	-0.404660	-0.101034
6	1	0	-2.023864	-0.258586	0.846719

Structure **$TS_{(9)+(8)\rightarrow(15)+(5)}$**

```
SCF Done:  E(UMN12SX) =  -453.910546
Zero-point correction=                           0.057514 (Hartree/Particle)
Thermal correction to Energy=                    0.064557
Thermal correction to Enthalpy=                  0.065501
Thermal correction to Gibbs Free Energy=         0.024832
Sum of electronic and zero-point Energies=           -453.853033
Sum of electronic and thermal Energies=              -453.845990
Sum of electronic and thermal Enthalpies=            -453.845046
Sum of electronic and thermal Free Energies=         -453.885715
```

One imaginary frequency: -1754.31i cm-1

Standard orientation:

Center Number	Atomic Number	Atomic Type	Coordinates (Angstroms) X	Y	Z
1	1	0	-1.932147	0.736257	0.009240
2	8	0	-2.801264	-0.100835	-0.081036
3	8	0	-0.801861	1.146258	0.025111
4	6	0	-0.206310	0.039353	-0.012821
5	8	0	-0.772927	-1.058434	-0.059587
6	1	0	-2.951794	-0.470715	0.808258
7	6	0	1.336479	0.088967	0.001939
8	8	0	1.930052	1.128971	-0.013121
9	8	0	1.855487	-1.113096	0.029751
10	1	0	2.827033	-1.058370	0.038850

Structure **15 + 5**

```
SCF Done:  E(UMN12SX) =  -453.928083822
Zero-point correction=                           0.059967 (Hartree/Particle)
Thermal correction to Energy=                    0.068687
Thermal correction to Enthalpy=                  0.069631
Thermal correction to Gibbs Free Energy=         0.025207
Sum of electronic and zero-point Energies=           -453.868117
Sum of electronic and thermal Energies=              -453.859397
Sum of electronic and thermal Enthalpies=            -453.858453
Sum of electronic and thermal Free Energies=         -453.902877
```

Standard orientation:

Center Number	Atomic Number	Atomic Type	Coordinates (Angstroms) X	Y	Z
1	1	0	-2.673880	0.677528	0.048622
2	8	0	-3.019423	-0.218684	-0.082389
3	8	0	-0.618640	1.333789	0.000302
4	6	0	-0.168438	0.215854	-0.012970
5	8	0	-0.761094	-0.909451	-0.040251
6	1	0	-3.118387	-0.584991	0.807085
7	6	0	1.397537	0.025972	0.003828
8	8	0	2.097094	0.991526	0.013234
9	8	0	1.762450	-1.227393	0.006551
10	1	0	2.734570	-1.301786	0.019561

Structure **$TS_{(15)+(8)\rightarrow(16)+(5)}$**

```
SCF Done:  E(UMN12SX) =  -453.214374
```

```
 Zero-point correction=                           0.042538 (Hartree/Particle)
 Thermal correction to Energy=                    0.049864
 Thermal correction to Enthalpy=                  0.050808
 Thermal correction to Gibbs Free Energy=         0.009121
 Sum of electronic and zero-point Energies=           -453.171836
 Sum of electronic and thermal Energies=              -453.164510
 Sum of electronic and thermal Enthalpies=            -453.163565
 Sum of electronic and thermal Free Energies=         -453.205253
```

One imaginary frequency: -1663.24i cm-1

Standard orientation:

Center Number	Atomic Number	Atomic Type	Coordinates (Angstroms) X	Y	Z
1	8	0	2.061757	-1.047932	0.019522
2	6	0	1.379522	-0.060213	0.004948
3	8	0	1.872319	1.139163	0.014105
4	6	0	-0.167764	-0.047342	-0.022882
5	8	0	-0.745164	-1.128658	-0.046565
6	8	0	-0.704161	1.089527	-0.009273
7	1	0	-1.899574	0.743783	-0.005769
8	8	0	-2.788501	-0.017042	-0.067231
9	1	0	-2.940989	-0.378919	0.828918

Structure **16**

```
 SCF Done:  E(UMN12SX) =  -376.832259
 Zero-point correction=                           0.021600 (Hartree/Particle)
 Thermal correction to Energy=                    0.027134
 Thermal correction to Enthalpy=                  0.028078
 Thermal correction to Gibbs Free Energy=        -0.008744
 Sum of electronic and zero-point Energies=           -376.810659
 Sum of electronic and thermal Energies=              -376.805126
 Sum of electronic and thermal Enthalpies=            -376.804181
 Sum of electronic and thermal Free Energies=         -376.841004
```

Standard orientation:

Center Number	Atomic Number	Atomic Type	Coordinates (Angstroms) X	Y	Z
1	8	0	-1.429060	-1.105140	-0.000010
2	6	0	-0.877266	-0.061381	0.000000
3	8	0	-1.132830	1.151174	0.000012
4	6	0	0.877260	-0.061384	-0.000002
5	8	0	1.429065	-1.105135	0.000010
6	8	0	1.132829	1.151175	-0.000012

Structure $\mathbf{TS}_{(16)\rightarrow 2(4)}$

```
 SCF Done:  E(UMN12SX) =  -376.830246
Zero-point correction=                           0.019915 (Hartree/Particle)
 Thermal correction to Energy=                    0.025417
 Thermal correction to Enthalpy=                  0.026361
 Thermal correction to Gibbs Free Energy=        -0.010863
 Sum of electronic and zero-point Energies=           -376.810331
 Sum of electronic and thermal Energies=              -376.804829
 Sum of electronic and thermal Enthalpies=            -376.803885
 Sum of electronic and thermal Free Energies=         -376.841109
```

One imaginary frequency: -279.46i cm-1

Standard orientation:

Center Number	Atomic Number	Atomic Type	Coordinates (Angstroms) X	Y	Z
1	8	0	-1.401289	-1.134514	-0.000003
2	6	0	-1.015132	-0.018344	-0.000002
3	8	0	-1.221908	1.159641	-0.000003
4	6	0	0.864024	-0.052438	0.000002
5	8	0	1.454902	-1.088597	0.000003
6	8	0	1.281626	1.116557	0.000003

Computational data for optimized structures at the UMN12SX/6-311++G(2d,p) level in gas phase

Structure **1**

```
SCF Done:  E(UMN12SX) =  -189.686685084
Zero-point correction=                           0.034362 (Hartree/Particle)
Thermal correction to Energy=                    0.037518
Thermal correction to Enthalpy=                  0.038462
Thermal correction to Gibbs Free Energy=         0.010307
Sum of electronic and zero-point Energies=           -189.652323
Sum of electronic and thermal Energies=              -189.649167
Sum of electronic and thermal Enthalpies=            -189.648223
Sum of electronic and thermal Free Energies=         -189.676378
```

Standard orientation:

Center Number	Atomic Number	Atomic Type	Coordinates (Angstroms) X	Y	Z
1	6	0	-0.132247	0.396570	0.000012
2	8	0	-1.124466	-0.263570	-0.000004
3	8	0	1.105615	-0.089229	-0.000006
4	1	0	-0.105245	1.499865	0.000043
5	1	0	1.049538	-1.056899	-0.000034

Structure **2**

```
SCF Done:  E(UMN12SX) =  -225.313788815
Zero-point correction=                           0.007771 (Hartree/Particle)
Thermal correction to Energy=                    0.010714
Thermal correction to Enthalpy=                  0.011658
Thermal correction to Gibbs Free Energy=        -0.015306
Sum of electronic and zero-point Energies=           -225.306017
Sum of electronic and thermal Energies=              -225.303075
Sum of electronic and thermal Enthalpies=            -225.302131
Sum of electronic and thermal Free Energies=         -225.329095
```

Standard orientation:

Center Number	Atomic Number	Atomic Type	Coordinates (Angstroms) X	Y	Z
1	8	0	-0.000000	0.000000	0.425148
2	8	0	0.000000	-1.066763	-0.212574
3	8	0	0.000000	1.066763	-0.212574

Structure **TS**$_{\mathbf{(1)+(2)\rightarrow(3)}}$

```
SCF Done:  E(UMN12SX) =  -414.972440323
Zero-point correction=                           0.036885 (Hartree/Particle)
Thermal correction to Energy=                    0.043731
Thermal correction to Enthalpy=                  0.044675
Thermal correction to Gibbs Free Energy=         0.004600
Sum of electronic and zero-point Energies=           -414.935555
Sum of electronic and thermal Energies=              -414.928710
Sum of electronic and thermal Enthalpies=            -414.927766
Sum of electronic and thermal Free Energies=         -414.967840
```

One imaginary frequency: -1837.22i cm-1

Standard orientation:

Center Number	Atomic Number	Atomic Type	Coordinates (Angstroms) X	Y	Z
1	6	0	1.226396	-0.154493	-0.024963
2	8	0	2.100948	-0.706303	0.544028
3	8	0	1.194154	1.058772	-0.526886
4	1	0	0.018480	-0.733332	-0.279385
5	1	0	2.039951	1.509907	-0.360354
6	8	0	-1.088473	-1.006178	-0.552568
7	8	0	-1.751889	0.161805	-0.291737
8	8	0	-1.631841	0.510702	0.925852

Structure **3**

```
SCF Done:  E(UMN12SX) =  -415.111753895
Zero-point correction=                           0.047603 (Hartree/Particle)
Thermal correction to Energy=                    0.053272
Thermal correction to Enthalpy=                  0.054216
Thermal correction to Gibbs Free Energy=         0.018190
Sum of electronic and zero-point Energies=           -415.064151
Sum of electronic and thermal Energies=              -415.058482
Sum of electronic and thermal Enthalpies=            -415.057538
Sum of electronic and thermal Free Energies=         -415.093564
```

Standard orientation:

Center Number	Atomic Number	Atomic Type	Coordinates (Angstroms) X	Y	Z
1	6	0	-0.939915	-0.054064	0.002734
2	8	0	-2.043254	-0.393419	0.278063
3	8	0	-0.542877	1.191830	-0.250018
4	1	0	1.574156	1.132115	0.467087
5	1	0	-1.327334	1.752037	-0.176392
6	8	0	1.921031	0.226136	0.525792
7	8	0	1.278998	-0.394250	-0.542529
8	8	0	0.060186	-0.950268	-0.049695

Structure $\mathbf{TS}_{(3)\rightarrow(4)+(5)+(6)}$

```
SCF Done:  E(UMN12SX) =  -415.060512
Zero-point correction=                           0.042340 (Hartree/Particle)
Thermal correction to Energy=                    0.047845
Thermal correction to Enthalpy=                  0.048789
Thermal correction to Gibbs Free Energy=         0.013035
Sum of electronic and zero-point Energies=           -415.018173
Sum of electronic and thermal Energies=              -415.012668
Sum of electronic and thermal Enthalpies=            -415.011723
Sum of electronic and thermal Free Energies=         -415.047478
```

One imaginary frequency: -740.53i cm-1

Standard orientation:

Center Number	Atomic Number	Atomic Type	Coordinates (Angstroms) X	Y	Z
1	1	0	2.038187	0.265758	1.059496
2	1	0	0.929074	1.028922	0.051456
3	8	0	1.878150	0.524998	0.138067
4	8	0	1.095565	-0.842793	-0.441032
5	8	0	0.029411	-1.021108	0.270566
6	8	0	-0.403587	1.225009	-0.159342
7	6	0	-1.073757	0.208594	-0.012006
8	8	0	-2.165129	-0.204387	0.061877

Structure **4**

```
SCF Done:  E(UMN12SX) =  -188.515403
Zero-point correction=                           0.011968 (Hartree/Particle)
Thermal correction to Energy=                    0.014569
Thermal correction to Enthalpy=                  0.015513
Thermal correction to Gibbs Free Energy=        -0.008705
Sum of electronic and zero-point Energies=           -188.503435
Sum of electronic and thermal Energies=              -188.500833
Sum of electronic and thermal Enthalpies=            -188.499889
Sum of electronic and thermal Free Energies=         -188.524108
```

Standard orientation:

Center Number	Atomic Number	Atomic Type	Coordinates (Angstroms) X	Y	Z
1	6	0	0.000000	0.000000	0.000000
2	8	0	0.000000	0.000000	1.153033
3	8	0	0.000000	0.000000	-1.153033

Structure **5**

```
SCF Done:  E(UMN12SX) =  -76.3953723260
Zero-point correction=                           0.021773 (Hartree/Particle)
Thermal correction to Energy=                    0.024608
Thermal correction to Enthalpy=                  0.025553
Thermal correction to Gibbs Free Energy=         0.004135
Sum of electronic and zero-point Energies=            -76.373599
Sum of electronic and thermal Energies=               -76.370764
Sum of electronic and thermal Enthalpies=             -76.369820
Sum of electronic and thermal Free Energies=          -76.391237

                        Standard orientation:
---------------------------------------------------------------------
Center     Atomic      Atomic             Coordinates (Angstroms)
Number     Number       Type             X           Y           Z
---------------------------------------------------------------------
     1          8           0        0.000000    0.000000    0.116346
     2          1           0        0.000000    0.765164   -0.465382
     3          1           0       -0.000000   -0.765164   -0.465382
---------------------------------------------------------------------
```

Structure **6**

```
SCF Done:  E(UMN12SX) =  -150.228011
Zero-point correction=                           0.003872 (Hartree/Particle)
Thermal correction to Energy=                    0.006235
Thermal correction to Enthalpy=                  0.007179
Thermal correction to Gibbs Free Energy=        -0.015044
Sum of electronic and zero-point Energies=           -150.224138
Sum of electronic and thermal Energies=              -150.221776
Sum of electronic and thermal Enthalpies=            -150.220832
Sum of electronic and thermal Free Energies=         -150.243055

                       Standard orientation:
---------------------------------------------------------------------
Center     Atomic      Atomic             Coordinates (Angstroms)
Number     Number       Type             X           Y           Z
---------------------------------------------------------------------
     1          8           0        0.000000    0.000000    0.599230
     2          8           0        0.000000    0.000000   -0.599230
---------------------------------------------------------------------
```

Structure **$TS_{(1)+(2)\rightarrow(7)}$**

```
SCF Done:  E(UMN12SX) =  -414.981202918
Zero-point correction=                           0.040020 (Hartree/Particle)
Thermal correction to Energy=                    0.045444
Thermal correction to Enthalpy=                  0.046388
Thermal correction to Gibbs Free Energy=         0.010766
Sum of electronic and zero-point Energies=           -414.941183
Sum of electronic and thermal Energies=              -414.935759
Sum of electronic and thermal Enthalpies=            -414.934815
Sum of electronic and thermal Free Energies=         -414.970437
```

One imaginary frequency: -1179.02i cm-1

```
                        Standard orientation:
---------------------------------------------------------------------
Center     Atomic      Atomic             Coordinates (Angstroms)
Number     Number       Type             X           Y           Z
---------------------------------------------------------------------
     1          1           0        0.240088    1.132154    0.104447
     2          1           0       -2.684234    0.135282   -0.125584
     3          8           0        1.383829    0.995799   -0.071637
     4          8           0        1.467703   -0.346778   -0.285312
     5          8           0        0.719920   -1.002735    0.457764
     6          8           0       -0.993476    1.168612    0.164863
     7          6           0       -1.579807    0.090343   -0.056269
     8          8           0       -1.087602   -1.041086   -0.220833
---------------------------------------------------------------------
```

Structure **7**

```
SCF Done:  E(UMN12SX) =  -414.997352124
Zero-point correction=                           0.044392 (Hartree/Particle)
Thermal correction to Energy=                    0.050690
Thermal correction to Enthalpy=                  0.051635
Thermal correction to Gibbs Free Energy=         0.013546
Sum of electronic and zero-point Energies=           -414.952960
Sum of electronic and thermal Energies=              -414.946662
Sum of electronic and thermal Enthalpies=            -414.945718
Sum of electronic and thermal Free Energies=         -414.983806

                        Standard orientation:
```

Center Number	Atomic Number	Atomic Type	X	Y	Z
1	1	0	-1.302080	1.068325	1.123446
2	1	0	2.264910	-0.254481	1.144853
3	8	0	-1.554718	1.019455	0.188375
4	8	0	-1.575758	-0.370827	-0.035320
5	8	0	-0.397297	-0.719561	-0.639946
6	8	0	1.679389	0.870289	-0.461082
7	6	0	1.563075	-0.000157	0.326647
8	8	0	0.555724	-0.900969	0.419451

Structure **TS**$_{\mathbf{(7)+(2)\rightarrow(4)+2(6)+2(8)}}$

```
SCF Done:  E(UMN12SX) =  -640.290587
Zero-point correction=                           0.047668 (Hartree/Particle)
Thermal correction to Energy=                    0.057411
Thermal correction to Enthalpy=                  0.058356
Thermal correction to Gibbs Free Energy=         0.011475
Sum of electronic and zero-point Energies=           -640.242919
Sum of electronic and thermal Energies=              -640.233176
Sum of electronic and thermal Enthalpies=            -640.232232
Sum of electronic and thermal Free Energies=         -640.279112
```

One imaginary frequency: -1736.33i cm-1

Standard orientation:

Center Number	Atomic Number	Atomic Type	X	Y	Z
1	1	0	-0.746363	-1.604018	0.598543
2	1	0	1.477273	1.000470	-0.115731
3	8	0	-1.641128	-1.265904	0.791728
4	8	0	-2.066655	-0.823461	-0.472851
5	8	0	-1.866720	0.508571	-0.534131
6	8	0	-0.164598	1.913598	1.031651
7	8	0	-0.407648	0.643003	-0.840870
8	8	0	2.498483	0.328777	-0.326537
9	8	0	1.998004	-0.916592	-0.431536
10	8	0	1.396941	-1.278459	0.636224
11	6	0	0.215942	1.287881	0.114628

Structure **8**

```
SCF Done:  E(UMN12SX) =  -75.7010582457
Zero-point correction=                           0.008667 (Hartree/Particle)
Thermal correction to Energy=                    0.011028
Thermal correction to Enthalpy=                  0.011972
Thermal correction to Gibbs Free Energy=        -0.008260
Sum of electronic and zero-point Energies=            -75.692391
Sum of electronic and thermal Energies=               -75.690030
Sum of electronic and thermal Enthalpies=             -75.689086
Sum of electronic and thermal Free Energies=          -75.709318
```

Standard orientation:

Center Number	Atomic Number	Atomic Type	X	Y	Z
1	8	0	0.000000	0.000000	0.108363
2	1	0	0.000000	0.000000	-0.866905

Structure **9**

```
SCF Done:  E(UMN12SX) =  -378.186648471
Zero-point correction=                           0.050231 (Hartree/Particle)
Thermal correction to Energy=                    0.055770
Thermal correction to Enthalpy=                  0.056714
Thermal correction to Gibbs Free Energy=         0.020174
Sum of electronic and zero-point Energies=           -378.136418
Sum of electronic and thermal Energies=              -378.130879
Sum of electronic and thermal Enthalpies=            -378.129935
Sum of electronic and thermal Free Energies=         -378.166474
```

Standard orientation:

Center Number	Atomic Number	Atomic Type	X	Y	Z
1	1	0	2.401755	0.703952	0.000029
2	8	0	1.465486	0.951199	0.000014

3	6	0	0.751573	-0.162716	-0.000020
4	8	0	1.191158	-1.270687	-0.000009
5	6	0	-0.751573	0.162716	-0.000014
6	8	0	-1.191158	1.270687	-0.000008
7	8	0	-1.465486	-0.951199	0.000020
8	1	0	-2.401755	-0.703952	0.000035

Structure $\mathbf{TS}_{(9)+(2)\rightarrow(10)}$

```
SCF Done:  E(UMN12SX) =  -603.481126345
Zero-point correction=                           0.055218 (Hartree/Particle)
Thermal correction to Energy=                    0.063614
Thermal correction to Enthalpy=                  0.064558
Thermal correction to Gibbs Free Energy=         0.020250
Sum of electronic and zero-point Energies=           -603.425908
Sum of electronic and thermal Energies=              -603.417513
Sum of electronic and thermal Enthalpies=            -603.416568
Sum of electronic and thermal Free Energies=         -603.460876
```

One imaginary frequency: -1039.44i cm-1

Standard orientation:

Center Number	Atomic Number	Atomic Type	Coordinates (Angstroms) X	Y	Z
1	1	0	1.372744	1.066030	0.368475
2	8	0	2.493812	1.013618	0.125273
3	8	0	2.621026	-0.246420	-0.368697
4	8	0	1.913981	-1.075370	0.219098
5	8	0	0.121186	1.052938	0.478631
6	6	0	-0.445785	0.046965	0.015068
7	8	0	0.050404	-0.996019	-0.434235
8	6	0	-1.990717	0.128566	-0.054726
9	8	0	-2.561272	1.076532	-0.487733
10	8	0	-2.544856	-0.979493	0.407727
11	1	0	-3.507986	-0.885516	0.348956

Structure **10**

```
SCF Done:  E(UMN12SX) =  -603.495499791
Zero-point correction=                           0.059830 (Hartree/Particle)
Thermal correction to Energy=                    0.068932
Thermal correction to Enthalpy=                  0.069877
Thermal correction to Gibbs Free Energy=         0.022850
Sum of electronic and zero-point Energies=           -603.435670
Sum of electronic and thermal Energies=              -603.426567
Sum of electronic and thermal Enthalpies=            -603.425623
Sum of electronic and thermal Free Energies=         -603.472650
```

Standard orientation:

Center Number	Atomic Number	Atomic Type	Coordinates (Angstroms) X	Y	Z
1	1	0	-1.966878	-1.007432	1.402114
2	8	0	-2.508125	-0.254916	1.115764
3	8	0	-2.529333	-0.436288	-0.281204
4	8	0	-1.609250	0.412958	-0.826287
5	8	0	0.374181	1.670191	0.269542
6	6	0	0.546938	0.557711	-0.082278
7	8	0	-0.341811	-0.261959	-0.681068
8	6	0	1.901273	-0.169960	0.063133
9	8	0	2.910843	0.429707	0.239170
10	8	0	1.780508	-1.489495	-0.008705
11	1	0	2.661511	-1.880664	0.095057

Structure $\mathbf{TS}_{(10)+(2)\rightarrow(11)}$

```
SCF Done:  E(UMN12SX) =  -828.793664
Zero-point correction=                           0.065157 (Hartree/Particle)
Thermal correction to Energy=                    0.077010
Thermal correction to Enthalpy=                  0.077954
Thermal correction to Gibbs Free Energy=         0.024537
Sum of electronic and zero-point Energies=           -828.728507
Sum of electronic and thermal Energies=              -828.716654
Sum of electronic and thermal Enthalpies=            -828.715710
Sum of electronic and thermal Free Energies=         -828.769126
```

One imaginary frequency: -926.54i cm-1

```
                        Standard orientation:
 ---------------------------------------------------------------------
 Center     Atomic      Atomic             Coordinates (Angstroms)
 Number     Number       Type             X           Y           Z
 ---------------------------------------------------------------------
      1          1           0        2.795228    0.868138    0.156898
      2          8           0        3.831538    0.479564   -0.104566
      3          8           0        3.596360   -0.832984   -0.358779
      4          8           0        2.739952   -1.330517    0.379253
      5          8           0        1.579416    1.216321    0.297685
      6          6           0        0.739413    0.342324    0.027941
      7          8           0        0.902375   -0.853361   -0.250678
      8          6           0       -0.732658    0.835884   -0.018363
      9          8           0       -1.089567    1.799518   -0.593453
     10          8           0       -1.487033   -0.040598    0.676396
     11          8           0       -2.857081    0.379261    0.684833
     12          8           0       -3.419085   -0.115806   -0.469496
     13          8           0       -3.778431   -1.451875   -0.224956
     14          1           0       -2.983315   -1.933571   -0.504289
 ---------------------------------------------------------------------
```

Structure **11**

```
 SCF Done:  E(UMN12SX) =  -828.810925
 Zero-point correction=                           0.070181 (Hartree/Particle)
 Thermal correction to Energy=                    0.082365
 Thermal correction to Enthalpy=                  0.083309
 Thermal correction to Gibbs Free Energy=         0.029151
 Sum of electronic and zero-point Energies=           -828.740744
 Sum of electronic and thermal Energies=              -828.728561
 Sum of electronic and thermal Enthalpies=            -828.727616
 Sum of electronic and thermal Free Energies=         -828.781774
```

```
                        Standard orientation:
 ---------------------------------------------------------------------
 Center     Atomic      Atomic             Coordinates (Angstroms)
 Number     Number       Type             X           Y           Z
 ---------------------------------------------------------------------
      1          1           0        3.403635    0.770736   -0.640333
      2          8           0        3.958548   -0.026494   -0.551842
      3          8           0        3.009666   -1.049365   -0.530637
      4          8           0        2.405813   -1.027056    0.735916
      5          8           0        1.648595    1.382411   -0.110385
      6          6           0        0.829918    0.576449    0.185612
      7          8           0        1.023220   -0.688881    0.551886
      8          6           0       -0.682622    0.911173    0.135823
      9          8           0       -1.078701    1.938725   -0.280418
     10          8           0       -1.405314   -0.118226    0.621023
     11          8           0       -2.798751    0.224993    0.639963
     12          8           0       -3.303758   -0.123119   -0.590394
     13          8           0       -3.641179   -1.485813   -0.538383
     14          1           0       -2.832531   -1.913850   -0.862117
 ---------------------------------------------------------------------
```

Structure **TS$_{(11)\rightarrow(4)+2(6)+2(8)}$**

```
 SCF Done:  E(UMN12SX) =  -828.771016
 Zero-point correction=                           0.067544 (Hartree/Particle)
 Thermal correction to Energy=                    0.079952
 Thermal correction to Enthalpy=                  0.080896
 Thermal correction to Gibbs Free Energy=         0.025760
 Sum of electronic and zero-point Energies=           -828.703473
 Sum of electronic and thermal Energies=              -828.691064
 Sum of electronic and thermal Enthalpies=            -828.690120
 Sum of electronic and thermal Free Energies=         -828.745256
```

One imaginary frequency: -258.90i cm-1

```
                        Standard orientation:
 ---------------------------------------------------------------------
 Center     Atomic      Atomic             Coordinates (Angstroms)
 Number     Number       Type             X           Y           Z
 ---------------------------------------------------------------------
      1          1           0        3.395747   -0.754150   -0.973774
      2          8           0        4.112333   -0.254645   -0.540640
      3          8           0        3.525201    0.125442    0.670909
      4          8           0        2.643802    1.168598    0.399066
      5          8           0        1.461313   -0.674374   -1.147195
      6          6           0        0.862339   -0.165106   -0.258786
      7          8           0        1.299181    0.694738    0.650972
      8          6           0       -0.647943   -0.482309    0.063950
      9          8           0       -0.844885   -1.390216    0.829764
     10          8           0       -1.402634    0.277211   -0.571887
     11          8           0       -3.203235   -0.805570    0.113483
     12          8           0       -3.820868    0.188006    0.507213
     13          8           0       -3.964236    1.129789   -0.516437
     14          1           0       -3.129898    0.966811   -1.019208
 ---------------------------------------------------------------------
```

Structure $TS_{(1)+(8)\rightarrow(12)+(5)}$

```
SCF Done:  E(UMN12SX) =  -265.384560544
Zero-point correction=                           0.041452 (Hartree/Particle)
Thermal correction to Energy=                    0.046686
Thermal correction to Enthalpy=                  0.047630
Thermal correction to Gibbs Free Energy=         0.012422
Sum of electronic and zero-point Energies=           -265.343109
Sum of electronic and thermal Energies=              -265.337875
Sum of electronic and thermal Enthalpies=            -265.336931
Sum of electronic and thermal Free Energies=         -265.372139
```

One imaginary frequency: -582.30i cm-1

Standard orientation:

Center Number	Atomic Number	Atomic Type	Coordinates (Angstroms) X	Y	Z
1	6	0	-0.449712	-0.165120	-0.000011
2	8	0	-1.308727	-0.980778	-0.000046
3	8	0	-0.608879	1.151394	0.000020
4	1	0	0.716749	-0.406435	0.000001
5	1	0	-1.558101	1.354665	0.000010
6	8	0	2.088713	-0.254563	0.000025
7	1	0	2.170773	0.714066	0.000062

Structure **12**

```
SCF Done:  E(UMN12SX) =  -189.019623825
Zero-point correction=                           0.021188 (Hartree/Particle)
Thermal correction to Energy=                    0.024368
Thermal correction to Enthalpy=                  0.025312
Thermal correction to Gibbs Free Energy=        -0.003219
Sum of electronic and zero-point Energies=           -188.998436
Sum of electronic and thermal Energies=              -188.995256
Sum of electronic and thermal Enthalpies=            -188.994312
Sum of electronic and thermal Free Energies=         -189.022843
```

Standard orientation:

Center Number	Atomic Number	Atomic Type	Coordinates (Angstroms) X	Y	Z
1	6	0	0.131452	-0.421080	-0.000040
2	8	0	1.146897	0.173930	0.000000
3	8	0	-1.106186	0.017759	0.000016
4	1	0	-1.114400	0.992970	0.000105

Structure **13**

```
SCF Done:  E(UMN12SX) =  -264.909833
Zero-point correction=                           0.040444 (Hartree/Particle)
Thermal correction to Energy=                    0.044161
Thermal correction to Enthalpy=                  0.045105
Thermal correction to Gibbs Free Energy=         0.014566
Sum of electronic and zero-point Energies=           -264.869389
Sum of electronic and thermal Energies=              -264.865672
Sum of electronic and thermal Enthalpies=            -264.864728
Sum of electronic and thermal Free Energies=         -264.895267
```

Standard orientation:

Center Number	Atomic Number	Atomic Type	Coordinates (Angstroms) X	Y	Z
1	6	0	-0.054440	-0.132615	0.000003
2	8	0	-0.744219	-1.101248	-0.000001
3	8	0	1.269632	-0.160550	0.000002
4	1	0	1.617100	0.740078	-0.000077
5	8	0	-0.503204	1.131636	0.000021
6	1	0	-1.468129	1.096908	-0.000125

Structure $TS_{(13)\rightarrow(4)+(5)}$

```
SCF Done:  E(UMN12SX) =  -264.834696
Zero-point correction=                           0.033976 (Hartree/Particle)
Thermal correction to Energy=                    0.037698
Thermal correction to Enthalpy=                  0.038643
Thermal correction to Gibbs Free Energy=         0.008007
```

```
 Sum of electronic and zero-point Energies=           -264.800719
 Sum of electronic and thermal Energies=              -264.796997
 Sum of electronic and thermal Enthalpies=            -264.796053
 Sum of electronic and thermal Free Energies=         -264.826688
```

One imaginary frequency: -1916.14i cm-1

Standard orientation:

Center Number	Atomic Number	Atomic Type	Coordinates (Angstroms) X	Y	Z
1	1	0	1.203537	-1.329653	0.625461
2	1	0	1.247648	0.472635	0.042234
3	8	0	1.067988	-0.707384	-0.101407
4	8	0	0.220787	1.216726	0.025083
5	6	0	-0.326913	0.091465	-0.009848
6	8	0	-1.349989	-0.470813	0.000247

Structure **TS$_{(1)+(8)\rightarrow(14)+(5)}$**

```
 SCF Done:  E(UMN12SX) =  -265.388856411
 Zero-point correction=                           0.042840 (Hartree/Particle)
 Thermal correction to Energy=                    0.047180
 Thermal correction to Enthalpy=                  0.048124
 Thermal correction to Gibbs Free Energy=         0.015583
 Sum of electronic and zero-point Energies=           -265.346017
 Sum of electronic and thermal Energies=              -265.341676
 Sum of electronic and thermal Enthalpies=            -265.340732
 Sum of electronic and thermal Free Energies=         -265.373274
```

One imaginary frequency: -1716.50i cm-1

Standard orientation:

Center Number	Atomic Number	Atomic Type	Coordinates (Angstroms) X	Y	Z
1	1	0	0.598147	-0.818512	0.008823
2	1	0	-2.096575	0.266451	-0.004607
3	8	0	1.567658	-0.105089	-0.099616
4	8	0	-0.573061	-1.069023	0.022858
5	6	0	-1.001665	0.110443	-0.001237
6	8	0	-0.277179	1.117412	-0.014644
7	1	0	1.769077	0.343001	0.734429

Structure **14**

```
 SCF Done:  E(UMN12SX) =  -188.997661
 Zero-point correction=                           0.017557 (Hartree/Particle)
 Thermal correction to Energy=                    0.020802
 Thermal correction to Enthalpy=                  0.021746
 Thermal correction to Gibbs Free Energy=        -0.006896
 Sum of electronic and zero-point Energies=           -188.980104
 Sum of electronic and thermal Energies=              -188.976859
 Sum of electronic and thermal Enthalpies=            -188.975915
 Sum of electronic and thermal Free Energies=         -189.004557
```

Standard orientation:

Center Number	Atomic Number	Atomic Type	Coordinates (Angstroms) X	Y	Z
1	1	0	-0.169770	1.374126	0.000001
2	8	0	1.152443	-0.161790	-0.000000
3	6	0	0.029135	0.230202	0.000001
4	8	0	-1.153074	-0.182628	-0.000000

Structure **TS$_{(14)+(8)\rightarrow(4)+(5)}$**

```
 SCF Done:  E(UMN12SX) =  -264.704147
 Zero-point correction=                           0.028599 (Hartree/Particle)
 Thermal correction to Energy=                    0.033192
 Thermal correction to Enthalpy=                  0.034137
 Thermal correction to Gibbs Free Energy=         0.000658
 Sum of electronic and zero-point Energies=           -264.675548
 Sum of electronic and thermal Energies=              -264.670954
 Sum of electronic and thermal Enthalpies=            -264.670010
 Sum of electronic and thermal Free Energies=         -264.703488
```

One imaginary frequency: -689.83i cm-1

Standard orientation:

Center Number	Atomic Number	Atomic Type	Coordinates (Angstroms) X	Y	Z
1	6	0	0.577599	-0.049285	-0.005631
2	8	0	1.690658	-0.464436	0.020750
3	8	0	-0.030582	1.044005	-0.022484
4	1	0	-0.451125	-0.800191	-0.014218
5	8	0	-1.787846	-0.432371	-0.091571
6	1	0	-1.992308	-0.081687	0.794444

Structure **TS$_{(9)+(8)\rightarrow(15)+(5)}$**

```
SCF Done:  E(UMN12SX) =  -453.888594
Zero-point correction=                           0.058331 (Hartree/Particle)
Thermal correction to Energy=                    0.065446
Thermal correction to Enthalpy=                  0.066390
Thermal correction to Gibbs Free Energy=         0.024919
Sum of electronic and zero-point Energies=           -453.830263
Sum of electronic and thermal Energies=              -453.823149
Sum of electronic and thermal Enthalpies=            -453.822204
Sum of electronic and thermal Free Energies=         -453.863675
```

One imaginary frequency: -1747.95i cm-1

Standard orientation:

Center Number	Atomic Number	Atomic Type	Coordinates (Angstroms) X	Y	Z
1	1	0	-1.925954	0.741275	0.048501
2	8	0	-2.787448	-0.095986	-0.108016
3	8	0	-0.804515	1.156168	0.090065
4	6	0	-0.214585	0.054484	0.004554
5	8	0	-0.777449	-1.044337	-0.072204
6	1	0	-2.939130	-0.600860	0.704539
7	6	0	1.332005	0.094697	-0.005428
8	8	0	1.946857	1.110020	-0.065075
9	8	0	1.841685	-1.124195	0.056723
10	1	0	2.807527	-1.048857	0.040274

Structure **15 + 5**

```
SCF Done:  E(UMN12SX) =  -453.904997311
Zero-point correction=                           0.060972 (Hartree/Particle)
Thermal correction to Energy=                    0.069536
Thermal correction to Enthalpy=                  0.070480
Thermal correction to Gibbs Free Energy=         0.025920
Sum of electronic and zero-point Energies=           -453.844025
Sum of electronic and thermal Energies=              -453.835462
Sum of electronic and thermal Enthalpies=            -453.834517
Sum of electronic and thermal Free Energies=         -453.879077
```

Standard orientation:

Center Number	Atomic Number	Atomic Type	Coordinates (Angstroms) X	Y	Z
1	1	0	-2.566110	0.659818	-0.035333
2	8	0	-2.997980	-0.204896	-0.117598
3	8	0	-0.667337	1.296767	0.039997
4	6	0	-0.182427	0.198297	0.010833
5	8	0	-0.735500	-0.950360	-0.008797
6	1	0	-3.176379	-0.504037	0.780227
7	6	0	1.389308	0.053977	-0.003563
8	8	0	2.086984	1.011073	-0.021824
9	8	0	1.782470	-1.207435	0.010022
10	1	0	2.752119	-1.230619	-0.002923

Structure **TS$_{(15)+(8)\rightarrow(16)+(5)}$**

```
SCF Done:  E(UMN12SX) =  -453.200328
Zero-point correction=                           0.043426 (Hartree/Particle)
Thermal correction to Energy=                    0.050660
Thermal correction to Enthalpy=                  0.051604
Thermal correction to Gibbs Free Energy=         0.010303
Sum of electronic and zero-point Energies=           -453.156901
Sum of electronic and thermal Energies=              -453.149668
Sum of electronic and thermal Enthalpies=            -453.148723
```

```
 Sum of electronic and thermal Free Energies=         -453.190025
```

One imaginary frequency: -1773.55i cm-1

Standard orientation:

Center Number	Atomic Number	Atomic Type	X	Y	Z
1	8	0	2.060148	-1.047516	-0.009087
2	6	0	1.370225	-0.071568	-0.002021
3	8	0	1.888124	1.115408	0.008347
4	6	0	-0.178121	-0.047224	0.000352
5	8	0	-0.787365	-1.111814	-0.005301
6	8	0	-0.692591	1.100078	0.016620
7	1	0	-1.857493	0.770168	0.006891
8	8	0	-2.765089	-0.002701	-0.100979
9	1	0	-2.920939	-0.485055	0.726323

Structure **16**

```
 SCF Done:  E(UMN12SX) =  -376.833965
 Zero-point correction=                           0.021628 (Hartree/Particle)
 Thermal correction to Energy=                    0.027260
 Thermal correction to Enthalpy=                  0.028204
 Thermal correction to Gibbs Free Energy=        -0.008816
 Sum of electronic and zero-point Energies=          -376.812337
 Sum of electronic and thermal Energies=             -376.806705
 Sum of electronic and thermal Enthalpies=           -376.805761
 Sum of electronic and thermal Free Energies=        -376.842781
```

Standard orientation:

Center Number	Atomic Number	Atomic Type	X	Y	Z
1	8	0	1.440749	-1.105152	-0.000086
2	6	0	0.893590	-0.061021	-0.000034
3	8	0	1.135091	1.150901	-0.000011
4	6	0	-0.893591	-0.061015	0.000030
5	8	0	-1.440767	-1.105137	0.000084
6	8	0	-1.135072	1.150915	0.000016

Structure $\mathbf{TS}_{(16)\rightarrow 2(4)}$

```
 SCF Done:  E(UMN12SX) =  -376.832655
 Zero-point correction=                           0.020417 (Hartree/Particle)
 Thermal correction to Energy=                    0.025876
 Thermal correction to Enthalpy=                  0.026820
 Thermal correction to Gibbs Free Energy=        -0.010195
 Sum of electronic and zero-point Energies=          -376.812238
 Sum of electronic and thermal Energies=             -376.806780
 Sum of electronic and thermal Enthalpies=           -376.805836
 Sum of electronic and thermal Free Energies=        -376.842850
```

One imaginary frequency: -345.94i cm-1

Standard orientation:

Center Number	Atomic Number	Atomic Type	X	Y	Z
1	8	0	1.444401	-1.131227	-0.000001
2	6	0	1.022143	-0.035089	0.000003
3	8	0	1.174123	1.156731	0.000006
4	6	0	-0.888263	-0.069891	-0.000001
5	8	0	-1.507884	-1.080085	0.000000
6	8	0	-1.211051	1.133316	-0.000007
